# Ecological theory of mutualism: Robust patterns of stability and thresholds in two‐species population models

**DOI:** 10.1002/ece3.8453

**Published:** 2021-12-15

**Authors:** Kayla R. S. Hale, Fernanda S. Valdovinos

**Affiliations:** ^1^ Department of Ecology and Evolutionary Biology University of Michigan Ann Arbor Michigan USA; ^2^ Department of Environmental Science and Policy University of California Davis California USA

**Keywords:** Allee effect, consumer‐resource, cost‐benefit, density‐dependence, density‐independence, dynamics, functional response, mutualism, overexploitation, stability, thresholds

## Abstract

Mutualisms are ubiquitous in nature, provide important ecosystem services, and involve many species of interest for conservation. Theoretical progress on the population dynamics of mutualistic interactions, however, comparatively lagged behind that of trophic and competitive interactions, leading to the impression that ecologists still lack a generalized framework to investigate the population dynamics of mutualisms. Yet, over the last 90 years, abundant theoretical work has accumulated, ranging from abstract to detailed. Here, we review and synthesize historical models of two‐species mutualisms. We find that population dynamics of mutualisms are qualitatively robust across derivations, including levels of detail, types of benefit, and inspiring systems. Specifically, mutualisms tend to exhibit stable coexistence at high density and destabilizing thresholds at low density. These dynamics emerge when benefits of mutualism saturate, whether due to intrinsic or extrinsic density dependence in intraspecific processes, interspecific processes, or both. We distinguish between thresholds resulting from Allee effects, low partner density, and high partner density, and their mathematical and conceptual causes. Our synthesis suggests that there exists a robust population dynamic theory of mutualism that can make general predictions.

## Graphical abstract

Theoretical progress on the ecology of mutualistic interactions comparatively lagged behind that of trophic and competitive interactions, leading to the impression that ecologists still lack a generalized framework to investigate the population dynamics of mutualisms. Yet, over the last 90 years, abundant work has accumulated, with qualitatively robust predictions across inspiring systems and levels of mechanistic detail. We review and synthesize this work, finding that mutualisms tend to exhibit stable coexistence at high density and destabilizing thresholds at low density.

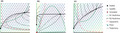

## 1 INTRODUCTION

Mutualisms are ubiquitous in nature and serve indispensable roles in supporting biodiversity and ecosystem function. Nearly all species on Earth participate in at least one of four main types of mutualism: seed dispersal, pollination, protection, and resource exchange including with symbionts (Bronstein, 2015a, 2015b; Janzen, 1985). Moreover, up to ~3/4 of phosphorus and nitrogen acquired by plants is provided by mycorrhizal fungi and nitrogen‐fixing bacteria (van der Heijden et al., 2008) and ~1/3 of crop production is dependent on animal pollination (Klein et al., 2007). The last 40 years has seen an important increase in studies on population ecology of mutualism but with no (e.g., Gotelli, 2008) to some representation in ecology textbooks (e.g., Kot, 2001; Mittelbach & McGill, 2019; Morin, 2011; Turchin, 2003; Vandermeer & Goldberg, 2013) and limited representation in recent syntheses of theoretical ecology (e.g., Hastings & Gross, 2012; but see McCann & Gellner, 2020 for a chapter on mutualistic networks by Bascompte and Ferrera, 2020). This historical underrepresentation of mutualisms in general ecology texts has been identified and explained by several authors (e.g., Boucher, 1985; Bronstein, 2015b; Raerinne, 2020), part of which we briefly describe below. We submit that ecology will benefit from integrating this coherent and robust body of theoretical work. Here, we contribute a first step toward such integration by presenting the ecological theory of mutualism available to the broader ecological community. Specifically, we review its historical literature and synthesize generalities, both mathematical and conceptual, that can lay a foundation for a deeper understanding and integration of mutualism in ecology.

Foundational theory in ecology was initially developed using Lotka–Volterra models. In this framework, constant coefficients describe the positive or negative effects between two interacting species as a linear function of the other species' density. The Lotka–Volterra model for predation and competition predict stable cycles (oscillations, Lotka, 1925; Volterra, 1926) and competitive exclusion (Gause, 1934; Volterra, 1926), respectively, which stimulated fruitful empirical and theoretical work. Indeed, from the groundwork of Lotka–Volterra theory of predation came more general consumer‐resource theory, with useful and surprising results such as the paradox of enrichment (Rosenzweig, 1971) and a mathematical representation of seasonal cycling in lake food webs (Boit et al., 2012).

In contrast, Lotka–Volterra models for mutualism have been a less useful simplification than for predation and competition (Holland, 2015). The original model (Gause & Witt, 1935) and other formulations in which species benefit as a linear function of each other's density (Addicott, 1981) can predict unbounded population growth of both species. Additionally, the diversity of mechanisms by which species may benefit each other and the non‐reciprocity of many of them, has cast suspicion on representing any “mutualistic” interaction as a simple exchange of positive effects (Bronstein, 2001a, 2001b). Mutualisms are more likely to exhibit shifting net effects than other interaction types (Chamberlain et al., 2014; Jones et al., 2015), with several exchanges dipping, for example, into parasitism.

Despite all these interesting mechanisms and patterns ripe for study, mutualisms have been subjected to less theoretical study than predation and competition. Many have speculated on historical reasons (Boucher, 1985; Bronstein, 2015b; Raerinne, 2020), but we highlight two here. First, the terms used to identify interactions as “mutualism” have changed over time. Previous theory treated mutualism as a subset of facilitation, in which one species alters the environment to benefit a neighboring species (Callaway, 2007), or symbiosis, in which species coexist in “prolonged physical intimacy” (Bronstein, 2015b), or used those terms interchangeably. Additionally, the terms “mutualism,” “cooperation,” and “protocooperation” have been used idiosyncratically for beneficial interactions within species as well as between them (Bronstein, 2015b). Furthermore, some mutualisms are “indirect,” such that benefits to one partner can only be realized in the presence of an external species or environmental condition (Holland & DeAngelis, 2010). In this review, we limit our scope to mutualism defined as reciprocally beneficial interactions between two species (Bronstein, 2015b). We largely focus on direct mutualism or models that approximate the effects of indirect mutualism through two‐species models, though we touch on some other cases (e.g., Thompson et al., 2006).

Second, the mechanisms by which species benefit each other in mutualisms are extremely diverse. These mechanisms include, but are not limited to, habitat provisioning, deterrence of predators or competitors, increased growth, faster maturation, facilitated reproduction, improved digestion, parasite grooming, and resource consumption. Conceptual frameworks have attempted to organize this rich diversity, for example, by the types of benefits exchanged (nutrition, protection, or transportation), the mechanisms of exchange, or the obligacy of each partner (reviewed in Bronstein, 2015b; Douglas, 2015). This diversity of mechanisms makes the development of general but informative theory for mutualism more difficult than, for example, predator‐prey theory, in which the interaction can be simply modeled as the consumption of individuals of one species by the individuals of the other species.

As it stands now, mutualism has repeatedly been called a loose set of natural history studies with little theory to unite or divide them (Addicott, 1981; Bronstein, 2015a). Despite an increasing number of theoretical studies, an “ecological theory of mutualism” has not penetrated the greater ecological community (Bronstein, 2015a; Gotelli, 2008; Kot, 2001; McCann & Gellner, 2020; Mittelbach & McGill, 2019; Morin, 2011; Turchin, 2003; Valdovinos, 2019; Vandermeer & Goldberg, 2013). The studies that exist have suffered from a pattern of neglect and repeated rediscovery (Boucher, 1985; Morin, 2011). Calls continue for simple but usable theory that synthesizes among mutualisms to identify patterns in population dynamics and in the mechanisms that generate them (e.g., Addicott, 1981; Bronstein, 1994, 2001a, 2015a; Callaway, 2007).

Here, we review ecological theory of mutualism, tracing authors' attempts to understand how mutualisms can persist stably overtime and synthesizing their results. We begin with an in‐depth historical review of the theoretical study of mutualism, highlighting many now‐obscure texts that have contributed to the field's current understanding. We focus exclusively on two‐species population‐dynamic models, leaving other aspects of historical mutualism research including game theory, biological market models, and eco‐evolutionary dynamics to previous (excellent) sources (Bronstein, 2015b; Hoeksema & Bruna, 2000). We organize the development of the theoretical study of mutualism semi‐chronologically, by its historical focus on the form of benefit either as linearly increasing with partner density or limited by intraspecific or interspecific density dependence, and its more recent incorporation into consumer‐resource theory (summarized in Table 1). After reviewing this rich and often overlooked body of work on the ecology of mutualism, we identify patterns in the predictions of these models that stand across systems and assumptions. In particular, we disentangle common terminology in order to clarify mechanisms that lead to predictable ecological dynamics. We find diverse, well‐characterized ecological mechanisms that permit stable coexistence. We additionally find that mutualisms are characterized by thresholds in density that may cause system collapse, which can be explained by partner dependence and interaction strength. We argue that extant models make a robust set of qualitative predictions and that these predictions qualify as an ecological theory of mutualism.

**TABLE 1:** The historical development of theory of mutualism

	Linear benefits	Saturating benefits (*intraspecific*)	Saturating benefits (*interspecific*)	Cost‐benefit models & shifting net effects	Consumer‐resource approach
Representative work	Gause and Witt (1935) proposed the first mutualism model as a modification of the Lotka–Volterra equations	Whittaker (1975) proposed that benefits to a host population from a symbiont should saturate per host individual due to extrinsic factors	Wright (1989) proposed that benefits should saturate with interspecific density, due to constraints on handling time	Hernandez (1998) proposed that benefits increase at low partner density, but interaction becomes negative at high partner density	Holland and DeAngelis (2010) proposed that resource supply and consumption processes directly affect per‐capita growth rate
Mechanisms included	Benefit increases per‐capita growth rate (low‐density effect), equilibrium density (high‐density effect), or both	Per‐capita benefit accrual decreases as: Resources or space become limiting*, Substrates to receive or attract benefits become limiting, Competition for benefits increases. * “extrinsic” factors; all other listed limitations are “intrinsic” to the mutualism	Rate of benefit accrual decreases as (effective) partner density becomes limiting, or due to satiation, search time, or handling time. Benefits may also be subject to intraspecific limitations	Partners have nonlinear effects, with positive effects (net benefits) at low recipient or partner densities and negative effects (net costs) at high densities. Benefits accrue due to facilitation at low density. Costs accrue due to exploitation or competition at high density	Benefits accrue due to consumption of resources (or services) supplied by a partner. Costs accrue by supplying resources to a partner or having resources consumed
Characteristic assumptions	Benefit is a linear function of partner density	Benefit increases per‐capita growth rate and equilibrium density, but saturates with increasing recipient density	Benefit increases per‐capita growth rate and equilibrium density, but saturates with increasing partner density. Recipient experiences additional self‐limitation	Net effects are represented directly as a non‐monotonic interspecific function or emerge from the balance between interspecific benefit and cost functions	Consumption is an interspecific process. Services are approximated as function of partner density or consumption rate. Costs accrue in demographic or foraging parameters (“fixed costs”), or are functions of partner consumption rate (“variable costs”)
Characteristic predictions	Unbounded growth between facultative partners with strong interactions. Stable coexistence between facultative partners with weak interactions. Extinction of obligate partners below a certain density threshold or unbounded growth above such threshold with strong interactions. Extinction of obligate partners with weak interactions	Stable coexistence in feasible interactions, regardless of interaction strength or obligacy. Threshold between extinction of obligate partners and stable coexistence when at least one partner is obligate. Coexistence is non‐oscillatory (stable node)	Same predictions as in intraspecific saturating models	Diverse dynamics, depending on the model and its parameterization: Predictions of saturating models, but coexistence may be oscillatory (stable spiral). Mutualistic coexistence, competitive coexistence, or competitive exclusion. Mutualistic coexistence, parasitic coexistence, or extinctions	Fixed costs: same predictions as in saturating models. Variable, linear costs: same predictions as saturating models, but coexistence may be oscillatory. Variable, nonlinear costs: mutualistic coexistence or overexploitation by consumers leading to collapse; coexistence may be oscillatory
Citations	Gause and Witt (1935), Whittaker (1975), Vandermeer and Boucher (1978), Goh (1979), Addicott (1981), Gilpin et al. (1982)	Whittaker (1975), May (1976), Soberón and Martinez del Rio (1981), Dean (1983), Wolin and Lawlor (1984), Parker (2001)	Wells (1983), Pierce and Young (1986), Wright (1989), Graves et al. (2006), Thompson et al. (2006), Fishman and Hadany (2010), Johnson and Amarasekare (2013), García‐Algarra et al. (2014)	Tonkyn (1986), Hernandez (1998), Holland et al. (2002), Neuhauser and Fargione (2004), Wu et al. (2019)	Holland and DeAngelis (2010), Kang et al. (2011), Revilla (2015), Martignoni et al. (2020), Hale et al. (2021)

## 2 HISTORICAL REVIEW

Mutualism research began with a simple Lotka–Volterra model in which per‐capita benefits increase linearly with partner density, which can lead to unbounded population growth or extinction (*Linear benefit models*, below). Since then a central organizing question has been, how can mutualisms persist over time without collapsing to extinction? Beginning in the 1970s, authors investigated mechanisms of inter‐ and intraspecific density dependency and mathematical forms that could cause benefits to saturate, limiting them from accumulating indefinitely (*Saturating benefit models*). As costs of participating in mutualisms were increasingly reported in empirical studies throughout the 1980s, the theory sought to understand if costs could account for limited net per‐capita benefits more mechanistically, as well as the conditions under which interactions could persist as mutualisms in light of context‐dependency of the net effects of the interaction (*Cost*‐*benefit models and shifting net effects*). Most recently, authors have sought to synthesize mutualism research into other bodies of interspecific ecological understanding, including consumer‐resource and ecological network theory (*Consumer*‐*resource approach to mutualism*).

**FIGURE 1:**
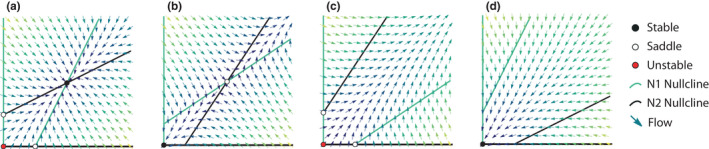
Characteristic dynamics for linear benefit models. In early models of mutualism, benefits were represented by a constant coefficient (interactions strength) multiplying a linear function of partner density. Benefits were modeled as affecting per‐capita growth rate (low‐density effect, Equation 4), equilibrium density (high‐density effect, Equation 2), or both (Equation 1, see Table 2). When benefits have exclusively low‐density effects, nullclines (curves of zero growth) are simply vertical ($N_{1}$) and horizontal ($N_{2}$) lines, always resulting in stable coexistence (qualitatively similar dynamics to those in a). Otherwise, the nullclines are linear, increasing curves, with different potential dynamics (a–d). When both partners are facultative mutualists ($N_{i}=K_{i}>0$ when $N_{j}=0$), they display stable coexistence when benefits are weak (a) or grow without bound (unstable coexistence) when benefits are strong (c). When both mutualists are obligate upon their partner ($N_{i}=K_{i}\leq 0$ when $N_{j}=0$) and benefits are weak, the system exhibits a threshold in density above which species exhibit unbounded growth and below which extinctions occur (b), whereas if benefits are strong, only extinctions occur (d). When mutualists are a facultative–obligate pair, any of the previous results can occur depending on relative interaction strength and obligacy. Benefit strength (weak or strong) is relative to intraspecific limitation. Arrows are vectors showing the “flow” of the system: arrow angle shows the direction of changes in density of $N_{1}$ (*x*‐direction) and $N_{2}$ (*y*‐direction) and arrow color shows the magnitudes of change in that direction (lighter colors are stronger changes). Nullclines are curves of zero change of density for one partner. Equilibria (colored or hollow dots) occur when both partners have zero change in density. Equilibria are locally stable (black dots) or unstable (red dots) if the system is attracted or repelled, respectively, the equilibrium after a small perturbation. Equilibria are half‐stable “saddles” (hollow dots) if the system is attracted in some dimensions by repelled in others. Panels were generated using the model in Case 1.1.1 of Table 3

**FIGURE 2:**
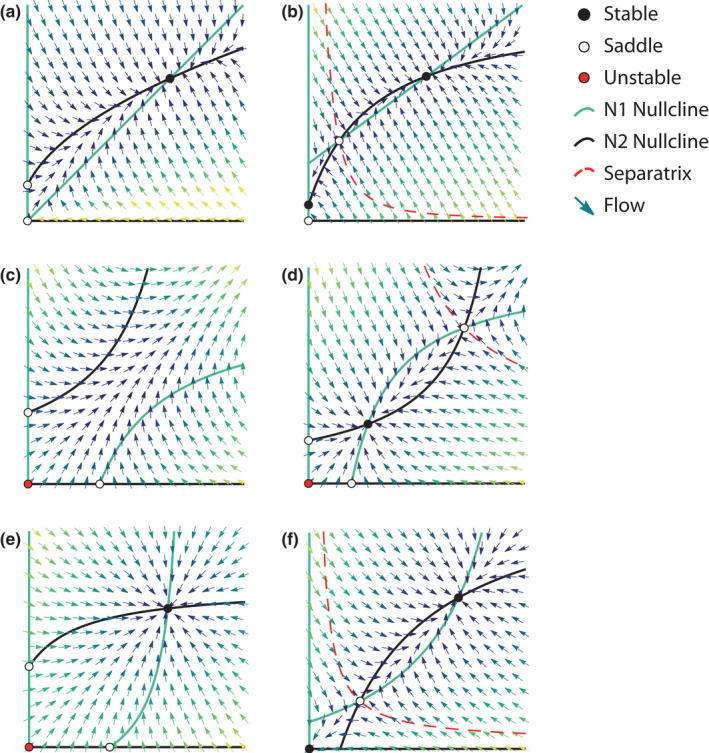
Characteristic dynamics for saturating benefit models. Density‐dependent benefit functions stabilize linear benefit models (Figure 1). Benefits may saturate (decrease in strength) with increasing recipient density (“intraspecific density‐dependence,” Case 2.1), increasing partner density (“interspecific density‐dependence,” Case 1.2), or both (Case 2.2), resulting in stable coexistence (see Table 3). Specifically, when paired with a partner with linear (a, b) or saturating (e, f) benefits, feasible systems exhibit the same qualitative dynamics: stable coexistence at densities higher than either partner could achieve alone (off‐axes black point), and potential or guaranteed threshold effects when one or both partners are obligate mutualists. Under a certain threshold (red dashed line), one population is at too low density to support its partner, collapsing the system (b, f). This threshold causes extinction of obligate partners, even if initially highly abundant (e.g., follow lighter colored trajectories in panel f). These dynamics of coexistence and threshold effects are robust across models of mutualism with saturating benefits, regardless of the mechanism by which benefit saturates (Cases 1.2, 2.1, 2.2). Benefits may also increase in strength with increasing recipient density (also called “intraspecific density‐dependence,” Case 3.2), causing unbounded growth in the absence of other limitations. Specifically, feasible systems between two facultative partners of this form exhibit unstable coexistence (c, d) and a potential threshold under which the system exhibits stable coexistence at low density or explodes with unbounded population growth at high density (d). Panels were generated using models in Case 1.1.1 ($N_{1}$ only, a, b), Case 1.2 for ($N_{2}$ only a‐b, both e‐f), or Case 3.2 (c, d) of Table 3

**FIGURE 3:**
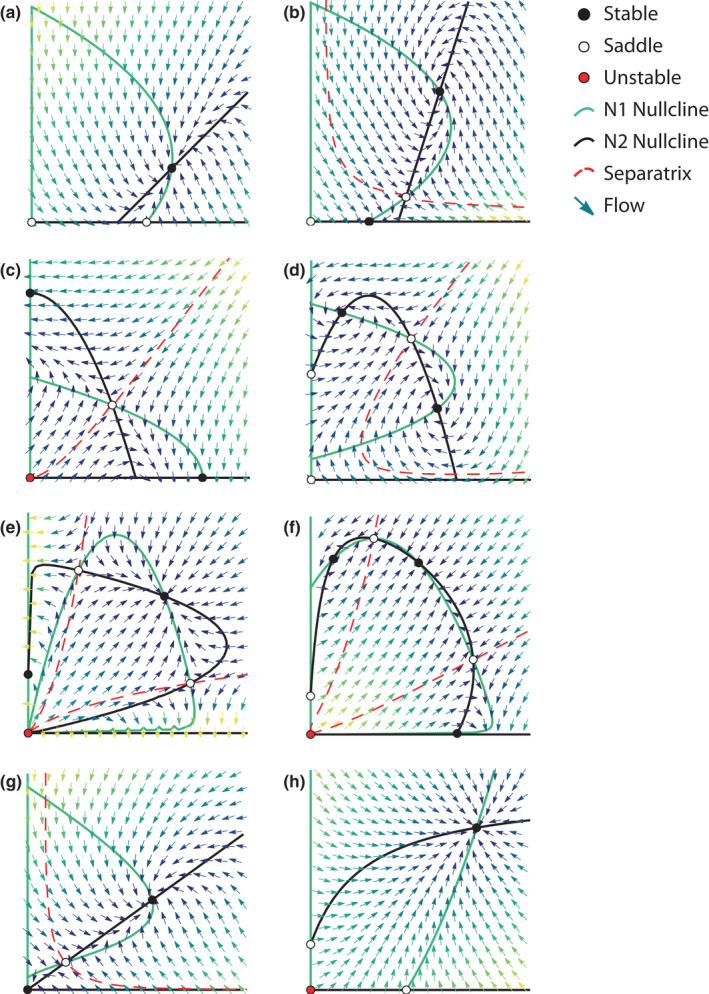
Characteristic dynamics for shifting net effects and consumer‐resource models. Models that investigated shifts in net effects as a balance of costs and benefits (“context‐dependency”) led to a synthesis of mutualism into a consumer‐resource framework. Models with saturating benefit functions and linear costs (a‐b) tend to display stable coexistence (a) and threshold effects (b) like earlier models (Figure 2). Stable coexistence is “mutualistic” if the nullclines intersect such that both species achieve higher density than they would alone, or if increasing the density of one species from equilibrium permit growth of its partner. Otherwise, the interaction is “parasitic.” Linear costs can make the coexistence equilibrium a stable spiral, with damped oscillations toward equilibrium (b, d, f, g). Models with unimodal benefit response that allow negative effects (net costs) at high density (c, d) or that include both separately saturating costs and benefits (e, f) display more complex dynamics. Depending on its parameterization, the mutualism‐competition model by Zhang (2003) displays mutualistic stable coexistence (not shown), competitive exclusion (c), or competitive dominance (d), with dominant species dependent on initial densities (i.e., system initialized to the left or right of the separatrix). The consumer‐resource model by Holland and DeAngelis (2010) also displays a range of dynamics depending on parameterization (e, f), including multiple stable coexistence equilibria (f). Mutualistic coexistence occurs when the ratio of consumers to their resources is not above a certain threshold (i.e., to the left of the left separatrix, or below the bottom separatrix). Otherwise, consumers overexploit their resources (causing more costs than provided benefits), leading to system collapse. Recent works use a consumer‐resource approach with system‐specific mechanisms (g, h), but often exhibit the simpler qualitative dynamics of saturating benefit models (Figure 2) with the potential for oscillations (g). Panels show the following models: (a–b) Neuhauser & Fargione, 2004, plant‐mycorrhizae; (c, d) Zhang, 2003, competitor‐mutualists; (e, f) Holland & DeAngelis, 2010, bidirectional consumer‐resource mutualism (e.g., corals); (g) Kang et al. 2011, ant‐fungal garden; (h) Hale et al. 2021, plant‐seed disperser

**FIGURE 4:**
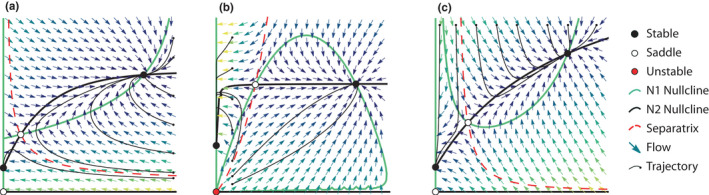
Distinguishing characteristic dynamics. $N_{1}$ (*x*‐axis) is obligate mutualist and $N_{2}$ (*y*‐axis) is facultative in all panels. (a) Threshold effects: $N_{1}$ goes extinct when the density of $N_{2}$ is below a threshold (separatrix). The system achieves stable coexistence when $N_{2}$ is above the threshold, and both species achieve higher densities than either would attain alone. (b) Overexploitation dynamics: the system collapses above a threshold in the ratio of consumer ($N_{2}$) to resource ($N_{1}$) species density. At low density, both partners will grow due to benefits from mutualism until they reach stable coexistence at higher density than either species could achieve alone. Above a threshold of $N_{2}$ density (separatrix), both populations will grow but $N_{2}$ will increase to such an extent that it exerts more costs than benefits it provides (exploitation). $N_{1}$ will begin to decline at low density while $N_{2}$ continues to grow, eventually leading to both going extinct. At even higher initial densities of $N_{2}$, $N_{2}$ will immediately overexploit $N_{1}$ and both species will go extinct, without even acquiring enough benefits to allow its own population to grow. (c) Allee effects: $N_{1}$ will go extinct if its density is under a threshold of its own density (left side of $N_{1}$ non‐trivial nullcline) because it becomes too rare to receive benefits from the mutualistic interaction. The system tends toward stable coexistence at higher density than either partner could achieve alone when $N_{1}$ is above a threshold of its own density (separatrix). Note that threshold effects induced by partner decline (a) cause Allee effects in both species because at low density they cannot support a sufficient partner population density to allow their own population growth. Overexploitation (b) by the high‐density consumer ($N_{2}$) also induces an Allee effect in the resource species ($N_{1}$) where lower resource density causes lower benefits from the interaction. Example systems: (a) Graves et al. 2006, lichens; (b) Holland & DeAngelis, 2010, unidirectional consumer resource mutualism (e.g., seed dispersal); (c) Hale et al. 2021, pollination

Below, we provide an in‐depth description of this theoretical development (summarized in Table 1). We focus on the bulk of theory that conforms to the typical assumptions of population dynamic approaches (Gotelli, 2008). That is, we focus on models without immigration or emigration (i.e., closed systems), without age, stage, or genetic structure, and under the approximation that individuals encounter each other randomly with no spatial structure (mean field assumption). These models have tended toward increasing analytical complexity as authors included more ecological mechanisms and system‐specific realism (Table 2), leveraging numerical equation solvers. Accordingly, we use phase plane diagrams (Figures 1, 2, 3, 4) to visualize the different qualitative dynamics of these models, as determined by species' curves of zero growth (“nullclines”) and fixed points (“equilibria”) of the system (summarized in Table 3).

**TABLE 2:** Selected models of pairwise mutualism

Reference	Eqn	Models for Pairwise Mutualism (i=1,2)	Notes
Gause and Witt (1935)	1	$\frac{dN_{i}}{\mathrm{dt}}=r_{i}N_{i}\left(\frac{K_{i}+\alpha_{\mathrm{ij}}N_{j}‐N_{i}}{K_{i}}\right)$	Facultative only
Whittaker (1975)	2 1	$\left\{\begin{array}{c} \frac{dN_{1}}{\mathrm{dt}}=r_{1}N_{1}\left(\frac{K_{1}+\alpha_{12}N_{2}‐N_{1}}{K_{1}+\alpha_{12}N_{2}}\right)\\ \frac{dN_{2}}{\mathrm{dt}}=r_{2}N_{2}\left(\frac{K_{2}+\alpha_{21}N_{1}‐N_{2}}{K_{2}}\right) \end{array}\right.$	Symbiont ($N_{1}$)‐Host ($N_{2}$) Obligate $N_{1}$ when $K_{1}=0$ Parasitism when $\alpha_{21}<0$
	2 3	$\left\{\begin{array}{c} \frac{dN_{1}}{\mathrm{dt}}=r_{1}N_{1}\left(\frac{\alpha_{12}N_{2}‐N_{1}}{\alpha_{12}N_{2}}\right)\\ \frac{dN_{2}}{\mathrm{dt}}=\frac{r_{2}N_{2}}{K_{2}}\left(K_{2}+\frac{aDN_{1}}{D+N_{2}}‐N_{2}\right) \end{array}\right.$	Symbiont ($N_{1}$)‐Host ($N_{2}$) Obligate $N_{1}$
Vandermeer and Boucher (1978)	1	$\frac{dN_{i}}{\mathrm{dt}}=N_{i}\left(r_{i}+\alpha_{\mathrm{ij}}N_{j}‐\alpha_{\mathrm{ii}}N_{i}\right)$	Legume ($N_{1}$)‐*Rhizobium* ($N_{2}$) Obligate when $K_{i}=r_{i}/\alpha_{\mathrm{ii}}\leq 0$
Addicott (1981)	4	$\frac{dN_{i}}{\mathrm{dt}}=r_{i}N_{i}\left(\frac{K_{i}‐N_{i}}{K_{i}}\right)\left(1+\frac{\alpha_{\mathrm{ij}}N_{j}}{K_{i}}\right)$	Aphid ($N_{1}$)‐Ant ($N_{2}$) Facultative only See Table S1
Wolin and Lawlor (1984)	5	$\frac{dN_{i}}{\mathrm{dt}}=N_{i}\left(r_{i}‐\frac{bN_{i}}{1+mN_{j}}‐dN_{i}\right)$	cultative only Reduces intra‐specific limitation in birth (b) to at most 0 See Table S1
	6	$\frac{dN_{i}}{\mathrm{dt}}=N_{i}\left(r_{i}‐\left(b‐mN_{j}+d\right)N_{i}\right)$	Reduces b without limit
Wright (1989)	7	$\frac{dN_{i}}{\mathrm{dt}}=N_{i}\left(r_{i}‐c_{i}N_{i}+b_{\mathrm{ij}}\frac{a_{\mathrm{ij}}N_{j}}{1+a_{\mathrm{ij}}h_{\mathrm{ij}}N_{j}}\right)$	Pollinators & other forager mutualists See Table S1
Zhang (2003)	8	$\frac{dN_{i}}{\mathrm{dt}}=R_{i}N_{i}\left(c_{i}‐N_{i}‐a_{i}{\left(N_{j}‐b_{i}\right)}^{2}\right)$	teractions between species at the same trophic level $‐\infty <b_{i}<\infty$
Neuhauser and Fargione (2004)	9 1	$\left\{\begin{array}{c} \frac{dN_{1}}{\mathrm{dt}}=r_{1}N_{1}\left(\frac{K_{1}+\gamma_{12}N_{2}‐N_{1}}{K_{1}+\gamma_{12}N_{2}}‐aN_{2}\right)\\ \frac{dN_{2}}{\mathrm{dt}}=r_{2}N_{2}\left(\frac{K_{2}+\alpha_{21}N_{1}‐N_{2}}{K_{2}}\right) \end{array}\right.$	Plant ($N_{1}$)‐Mycorrhizae ($N_{2}$) Facultative only
Graves et al. (2006)	10	$\frac{dN_{i}}{\mathrm{dt}}=N_{i}\left(r_{i0}+\left(r_{i1}‐r_{i0}\right)\left(1‐e^{‐k_{i}N_{j}}\right)‐a_{i}N_{i}\right)$	Lichens Obligate when $r_{i0}\langle 0,r_{i1}+r_{i0}\rangle 0$ See Table S1
Thompson et al. (2006)	11 12	$\left\{\begin{array}{c} \frac{dN_{1}}{\mathrm{dt}}=\left(\rho_{1}b_{1}N_{1}+I_{1}\right)\left(1‐\frac{N_{1}}{S_{1}}\right)‐\left(d_{1_{\text{min}}}+\frac{d_{1_{\text{max}}}‐d_{1_{\text{min}}}}{1+c_{1}N_{2}}\right)N_{1}\\ \frac{dN_{2}}{\mathrm{dt}}=\left(\rho_{2}b_{2}N_{2}+I_{2}\right)\left(1‐\frac{N_{2}}{S_{2}+N_{1}}\right)‐\left(d_{1_{\text{min}}}+\frac{d_{2_{\text{max}}}‐d_{2_{\text{min}}}}{1+c_{2}N_{1}}\right)N_{2} \end{array}\right.$	Hermit crabs ($N_{1}$)‐ Anemones ($N_{2}$) Closed system when $I_{i}=0$, $\rho_{i}=1$ Obligate when $\rho_{i}b_{i}<d_{i_{\max}}$ See Table S1
Holland and DeAngelis (2010)	13	$\frac{dN_{i}}{\mathrm{dt}}=N_{i}\left(r_{i}+c_{i}\left(\frac{a_{\mathrm{ij}}N_{j}}{h_{j}+N_{j}}\right)‐q_{i}\left(\frac{\beta_{\mathrm{ij}}N_{j}}{e_{i}+N_{i}}\right)‐s_{i}N_{i}\right)$	Bidirectional Consumer‐Resource e.g., Plant ($N_{1}$)‐Mycorrhizae ($N_{2}$) Obligate when $r_{i}=0$
	13 7	$\left\{\begin{array}{c} \frac{dN_{1}}{\mathrm{dt}}=N_{1}\left(r_{1}+c_{1}\left(\frac{a_{12}N_{2}}{h_{2}+N_{2}}\right)‐q_{1}\left(\frac{\beta_{12}N_{2}}{e_{1}+N_{1}}\right)‐s_{1}N_{1}\right)\\ \frac{dN_{2}}{\mathrm{dt}}=N_{2}\left(r_{2}+c_{2}\left(\frac{a_{21}N_{1}}{h_{1}+N_{1}}\right)‐s_{2}N_{2}\right) \end{array}\right.$	Unidirectional e.g., Plant ($N_{1}$)‐Pollinator ($N_{2}$)
Fishman and Hadany (2010)	14 15	$\left\{\begin{array}{c} \frac{dN_{1}}{\mathrm{dt}}=N_{1}\left(\frac{\eta \alpha N_{2}}{1+\alpha N_{1}+\alpha \beta N_{2}}‐b‐cN_{1}\right)\\ \frac{dN_{2}}{\mathrm{dt}}=N_{2}\left(\frac{\mu \alpha N_{1}}{1+\alpha N_{1}+\alpha \beta N_{2}}‐d\right) \end{array}\right.$	Plant ($N_{1}$)‐Pollinator ($N_{2}$) Obligate only
Kang et al. (2011)	16 1	$\left\{\begin{array}{c} \frac{dN_{1}}{\mathrm{dt}}=N_{1}\left(r_{f}\left(\frac{aN_{2}^{2}}{b+aN_{2}^{2}}\right)‐r_{c}N_{2}‐d_{1}N_{1}\right)\\ \frac{dN_{2}}{\mathrm{dt}}=N_{2}\left(r_{a}N_{1}‐d_{2}N_{2}\right) \end{array}\right.$	Fungal garden ($N_{1}$)‐Leaf cutter ant ($N_{2}$) Obligate only
Martignoni et al. (2020)	17 18	$\left\{\begin{array}{c} \frac{dN_{1}}{\mathrm{dt}}=N_{1}\left(r_{p}+\frac{q_{\mathrm{hp}}\alpha N_{2}}{d+N_{1}}‐q_{\mathrm{cp}}\beta N_{2}‐\mu_{p}N_{1}\right)\\ \frac{dN_{2}}{\mathrm{dt}}=N_{2}\left(q_{\mathrm{cm}}\beta N_{1}‐\frac{q_{\mathrm{hm}}\alpha N_{1}}{d+N_{1}}‐\mu_{m}N_{2}\right) \end{array}\right.$	Plant ($N_{1}$)‐Mycorrhizae ($N_{2}$) Obligate $N_{2}$
Hale et al. (2021)	19 7	$\left\{\begin{array}{c} \frac{dP}{\mathrm{dt}}=P\left[b_{P}\left(f+\varphi \frac{a\mathrm{AP}}{1+ahP+a\mathrm{AP}}\right)g‐s_{P}P‐d_{P}\right]\\ \frac{dA}{\mathrm{dt}}=A\left[b_{A}+\varepsilon \frac{aP}{1+ahP}‐s_{A}A‐d_{A}\right] \end{array}\right.$	Plant ($N_{1}$)‐Pollinator ($N_{2}$) Obligate $N_{1}$ when $b_{1}fg‐d_{1}\leq 0$; obligate $N_{2}$ when $b_{2}‐d_{2}\leq 0$
	20 7	$\left\{\begin{array}{c} \frac{dP}{\mathrm{dt}}=P\left[b_{P}fg‐\left(s_{P}‐\sigma \frac{aA}{1+ahP+aA}\right)P‐d_{P}\right]\\ \frac{dA}{\mathrm{dt}}=A\left[b_{A}+\varepsilon \frac{aP}{1+ahP}‐s_{A}A‐d_{A}\right] \end{array}\right.$	Plant ($N_{1}$)‐Seed Disperser ($N_{2}$) Facultative $N_{1}$ only Obligate $N_{2}$ when $b_{2}‐d_{2}\leq 0$

Note

A full list of models cited in the main text is included in the supplementary information (Table S1). Equations largely follow the notation from the original citations. All parameters are positive (>0) unless otherwise specified. Models with unique mathematical forms are given unique equation numbers. We encourage the readers to refer to the original references for the model derivations and interpretation of parameters. Notes include inspiring system and obligacy, if specified by authors.

**TABLE 3:** Generic models of mutualism

	Change in population density of $N_{i}$ with benefits from $N_{j}$	$N_{1}$ Nullcline geometry	Qualitative dynamics with $N_{2}$		Empirical justification (Table S1 reference)
			Stability with Case 1.1.1 (linear)	Stability with Case 1.2 (increasing, concave down)	
Case 1: Intraspecific density dependence in population dynamics only: self‐limitation or negative density dependence terms					
	Benefits accrue directly to per‐capita growth rate…				
1.1.1	…as a linear function of partner density $\frac{dN_{i}}{\mathrm{dt}}=N_{i}\left(r_{i}+\beta_{\mathrm{ij}}N_{j}‐s_{i}N_{i}^{\theta_{i}}\right)$	$\theta_{1}=1$: Increasing, linear	SC (Figure 1a) UC (Figure 1c) UC/E threshold (Figure 1b)	SC (Figure 2a) HD SC & SC/E threshold (Figure 2b)	S1: Early ant colonies consume fungus, self‐limit due to larval care, etc. (Kang et al., 2011)
1.1.2		$0<\theta_{1}<1$, $r_{1}>0$: Increasing concave down	UC UC/E threshold HD UC & SC/E threshold	SC UC UC/E threshold HD UC & SC/E threshold	S35: Decelerating negative density‐dependence; “r‐selected” organisms (Moore et al., 2018)
1.1.3		$\theta_{1}>1$,$r_{1}>0$: Increasing, concave up	SC HD SC & SC/E threshold	SC (Figure 2e) HD SC & SC/E threshold (Figure 2f)	S35: Accelerating negative density‐dependence; “K‐selected,” sedentary, & stage‐structured organisms, e.g., flowering plants (Moore et al., 2018)
1.2	…as a function that saturates with increasing partner density $\frac{dN_{i}}{\mathrm{dt}}=N_{i}\left(r_{i}+\beta_{\mathrm{ij}}\frac{N_{j}}{h_{\mathrm{ij}}+N_{j}}‐s_{i}N_{i}\right)$	Increasing, concave up	SC HD SC & SC/E threshold	SC (Figure 2e) HD SC & SC/E threshold (Figure 2f)	Servicers such as pollinators forage… S8: limited by handling time (Type II, Hale et al., 2021; Holland & DeAngelis, 2010; Soberón & Martinez del Rio, 1981; Wright, 1989) S34: limited by rewards availability (Type I, on saturating plant rewards Revilla, 2015) S27: Mortality declines due to protection or deterrence by partners (Thompson et al., 2006)
Case 2: Intraspecific density‐dependence in mutualism only: benefits saturate with increasing recipient density					
	Benefits accrue directly to per‐capita growth rate…				
2.1	……with increasing recipient density $\frac{dN_{i}}{\mathrm{dt}}=N_{i}\left(r_{i}+\beta_{\mathrm{ij}}\frac{N_{j}}{h_{\mathrm{ij}}+N_{i}}‐s_{i}N_{i}\right)$	Increasing, concave up	SC HD SC & SC/E threshold	SC (Figure 2e) HD SC & SC/E threshold (Figure 2f)	Plant reproduction is a function of pollinator visitation… S7: Type II, on plants (Soberón & Martinez del Rio, 1981) S33: Type I, on saturating plant rewards (Revilla, 2015) Also see S3
2.2	……with increasing recipient & partner density $\frac{dN_{i}}{\mathrm{dt}}=N_{i}\left(r_{i}+\beta_{\mathrm{ij}}\frac{N_{j}}{h_{\mathrm{ij}}+N_{i}+N_{j}}‐s_{i}N_{i}\right)$	Increasing, concave up	SC HD SC & SC/E threshold	SC (Figure 2e) HD SC & SC/E threshold (Figure 2f)	S10: Plant reproduction is a function of pollinator visitation (Type II), limited by ovule availability (Wells, 1983) S11: Pollinators forage on plants (Type II), limited by search time (Wells, 1983) Also see S4, S31
Case 3: Benefits of mutualism reduce intraspecific density‐dependence in population dynamics					
	Benefits reduce negative density‐dependence…				
3.1	…via increasing carrying capacity as a linear function of partner density $\frac{dN_{i}}{\mathrm{dt}}=r_{i}N_{i}\left(1‐\frac{N_{i}}{K_{i}+N_{j}}\right)$	Increasing, linear	SC (Figure 1a) UC (Figure 1c) UC/E threshold (Figure 1b)	SC (Figure 2a) HD SC & SC/E threshold (Figure 2b)	S2: Hosts for symbionts (May, 1976; Whittaker, 1975) S28: Partners supply substrate or habitat, e.g., domatia for aphids (Thompson et al., 2006) Also see S12
	…via decreasing self‐limitation				
3.2	……as a linear function of partner density $\frac{dN_{i}}{\mathrm{dt}}=N_{i}\left(r_{i}‐\left(s_{i}‐\beta_{\mathrm{ij}}N_{j}\right)N_{i}\right)$	Increasing, concave down	UC HD UC & UC/SC threshold	UC HD UC & UC/SC threshold	S13: Benefits accrue primarily at high recipient density (Wolin & Lawlor, 1984)
3.3	……as a function that saturates with increasing recipient & partner density $\frac{dN_{i}}{\mathrm{dt}}=N_{i}\left(r_{i}‐\left(s_{i}‐\beta_{\mathrm{ij}}\frac{N_{j}}{h_{\mathrm{ij}}+N_{i}+N_{j}}\right)N_{i}\right)$	Decreasing, concave up to linear	SC HD SC & SC/E threshold	SC (Figure 3h) HD SC & SC/E threshold (Figure 3h)	S40: Disperser visitation (Type II) reduces seed mortality from the Janzen‐Connell effect (Hale et al., 2021)

Note

Description of nullcline geometry, qualitative dynamics, and empirical assumptions under which seven generic models of mutualism may arise. In all models, benefits of mutualism are a function of partner density ($N_{j}$). All models also include a form of intraspecific density dependence, that is per‐capita growth rate is dependent upon recipient density ($N_{i}$). To better interpret the historical literature, we categorize models into three cases of intraspecific density dependence (see text). Only Case 2 yields feasible dynamics in the absence of self‐limitation (i.e., when $s_{i}=0$). Intrinsic (per‐capita) growth rate determines obligacy in all models ($r_{i}\leq 0$: i is obligate upon j, $r_{i}>0$: i is facultative), with one exception. Case 3.1 uses the (deprecated) historical convention in which carrying capacity directly determines obligacy ($K_{i}=0$: i is obligate upon j, $K_{i}>0$: i is facultative). All other parameters are assumed to be positive. Nullcline geometry is restricted to the ecologically relevant region ($N_{1}$≥0, $N_{2}$≥0). Only feasible dynamics are listed: “SC” is stable coexistence, “UC” is unstable coexistence,” “UC/E threshold” is a threshold dividing the plane into unstable coexistence at higher density or extinction at lower density, “HD” is high density, etc. Alternative qualitative dynamics (listed on separate lines) are possible based on parameterization of the models.

### 2.1 Linear benefit models

Gause and Witt (1935) proposed a model for “mutual aid” between a host and symbiont, inspired by Konstitzin (1934; Wolin, 1985). This model was a modification of the Lotka–Volterra competition equations with positive (instead of negative) interaction coefficients (Equation 1; see Table 2 for numbered equations). Benefits increased linearly with increasing partner density, while the strength of negative (intraspecific) density dependence arising from processes external to the mutualism also increased linearly with the density of the species receiving the benefit (i.e., the recipient species; Figure 1). In this formulation, mutualism has two effects: it increases the low‐density growth rate of the recipient and the highest density at which the recipient can persist (typically, the equilibrium density). The second effect has been called an increase in “carrying capacity,” but we reserve that term for density in the absence of the mutualistic partner. As written, the model accommodates only what we now call “facultative” mutualists (Vandermeer & Boucher, 1978), those that can persist at positive density (“carrying capacity”, K) in the absence of their partner (K>0). Gause and Witt also commented that increasing the strength of mutualism ($\alpha_{\mathrm{ij}}$, Equation 1) increases both species' equilibrium biomass until they pass to infinity, but that infinite populations are obviously unreasonable and microcosm studies suggest that interaction strength should decrease as species grow. These two studies (i.eGause & Witt, 1935; Kostitzin, 1934) initiated theoretical research on what we now call mutualism around the same time as theoretical research on predation and competition, but then paused for nearly 40 years.

Beginning in the 1970s, mutualism received attention as a destabilizing force in ecological networks represented as random community matrices (May, 1972, 1973), with the unbounded growth in the Lotka–Volterra models of mutualism being called a “silly solution” (May, 1976). Using Lotka–Volterra models, authors better characterized the conditions that lead to unbounded growth found by Gause and Witt's original model of mutualism (Albrecht et al., 1974; Goh, 1979; Travis & Post, 1979; Vandermeer & Boucher, 1978). Other forms of linear benefits were investigated such as those that increase per‐capita growth rate, equilibrium density, or both (Figure 1). Whittaker (1975) introduced a model in which mutualism increases the equilibrium density of one partner and both the equilibrium density and per‐capita growth rate of the other partner. This model accommodates “obligate” mutualists like symbionts living on a host that cannot persist in the absence of that host, that is, have zero carrying capacity (K=0) in the absence of their partners. The mutualistic symbiont–host interaction linearly increases the carrying capacity for the symbiont (Equation 2) while benefiting the host population by increasing its low‐density growth rate and its equilibrium density (Equation 1). Later, Addicott (1981) introduced a model in which mutualism only increases the per‐capita growth rate (Equation 4), inspired by the ant‐aphid mutualism described in Addicott (1979). Addicott emphasized that these different linear benefit models could be used in a mix‐and‐match style to accommodate different types of benefits exchanges.

Vandermeer and Boucher (1978) proposed the groundbreaking idea that mutualistic partners may exist along continuums of obligacy and interaction strength. The authors defined facultative mutualists as those with positive carrying capacity in absence of their partner. Obligate mutualists were defined more abstractly with zero or negative carrying capacity in absence of their partner (K≤0), which represents the demographic drawdown that mutualism must exceed to allow persistence of the population. Negative carrying capacity arises mathematically when a population has a negative “intrinsic” growth rate, as is the case when its per‐capita death rate exceeds its per‐capita birth rate ($K_{i}=r_{i}/a_{\mathrm{ii}}<0$, where $a_{\mathrm{ii}}>0$ is a self‐limitation coefficient, Table 2). This choice is useful both mathematically and ecologically because it allows the strong demographic pulldown when death rates exceed birth rates to be represented, without introducing numerical issues due to zero carrying capacity. Vandermeer and Boucher's analysis of Gause and Witt (1935)'s model found that obligate–obligate partnerships would either collapse to extinction when benefits are weak or exhibit a threshold population size under which they go extinct and above which they grow unboundedly when benefits are strong (Figure 1b,d). They also found that facultative partners are likely to coexist stably when benefits are weak or exhibit unbounded growth when benefits are strong (Figure 1a,c, also see Wolin, 1985). Remarkably, Vandermeer and Boucher (1978; also see Christiansen & Fenchel, 1977) anticipated the qualitative dynamics generated by extending these models to saturating benefit responses. However, the authors emphasized that unbounded growth was still an ecologically relevant result because it indicates persistence of the two‐species mutualistic system. Indeed, they argue that persistence (whether species persist or go extinct) is a more biologically useful metric than neighborhood stability (whether the system returns to equilibrium after a small perturbation). Subsequent authors also emphasized other properties of stability of mutualism such as return time to equilibrium (Addicott, 1981; Wolin, 1985), the domain of attraction to equilibrium (Benadi et al., 2013a), species persistence (Valdovinos et al., 2013, 2016, 2018), maintenance of diversity (Benadi et al., 2013b), and biomass variability (Hale et al., 2020).

### 2.2 Saturating benefit models

The earliest models that incorporated saturating benefits within mutualism invoked unspecified (intraspecific) environmental constraints that limit population growth in the presence of a mutualist (Dean, 1983; May, 1976; Whittaker, 1975; Wolin & Lawlor, 1984). For example, Whittaker (1975) assumed extrinsic, intraspecific limiting factors to the benefits a host could receive from its symbiont (Equation 3, Figure 2a). This is the first of many models that exhibit thresholds (*sensu* Vandermeer & Boucher, 1978), where the low density of one partner pushes the whole system to collapse (sometimes called “Allee thresholds,” e.g., Johnson & Amarasekare, 2013).

This focus on extrinsic limits to benefit was epitomized by Wolin and Lawlor (1984). They derived models for five different ways in which mutualism could affect per‐capita birth or death rates as functions of recipient density. For example, they compared models in which mutualism reduces intraspecific density‐dependent limiting factors only in per‐capita birth rates (Equation 6, Figure 2c,d), only in per‐capita birth rate but with saturating effects (Equation 5, Figure 2e), and both in birth and death rates with saturating effects (Equation 2, Figure 1a). These models were classified as describing mutualisms with effects primarily at high versus low self‐density. Wolin and Lawlor concluded that low‐density effects (i.e., primary effects on per‐capita growth rate as opposed to equilibrium density) are stabilizing in terms of faster return times and the existence of a feasible, stable equilibrium. These models of “intraspecific density‐dependence” (so‐called by later authors, Holland, 2015) lacked biological mechanisms or reference to clear ecological examples, which perhaps pivoted the field away from this otherwise fruitful approach. In contrast, Soberón and Martinez del Rio (1981) proposed a detailed pollination model in which plant benefits are a function of pollinators' visitation rate, modeled as a saturating Type II functional response. Thus, benefits to plants saturate as a function of their own density (intraspecific density dependence), but due to factors intrinsic to the mutualism (that is, time constraints for pollinators handling flowers during foraging visits). Such an approach has seen a resurgence in recent literature (see *Consumer*‐*resource approach*, below) but was largely abandoned at the time.

Starting in the late 1980s, authors began to focus on “interspecific density dependence,” which has been considered more consistent with other theories of interspecific interactions (Holland, 2015). Wright (1989) proposed a model in which per‐capita benefits saturate in terms of partner density analogously to consumers foraging on resources due to limitations of consumer handling of resources or uptake rate (Figure 2e,f). In the mutualistic case, benefits are assumed to saturate with increasing partner density, often as an additive, first‐order term to per‐capita growth rate following a Holling Type II functional response (Bazykin, 1997; Hale et al., 2021; Holland & DeAngelis, 2010; Thompson et al., 2006; Wright, 1989; Wu et al., 2019). On the other hand, Thompson et al. (2006) proposed a theoretical framework that organized both terrestrial and aquatic mutualisms into those that affect birth rate, death rate, habitat acquisition, or a combination of these benefits for each partner. Other authors have used different mathematical forms for analytical tractability (García‐Algarra et al., 2014; Pierce & Young, 1986). Regardless, these assumptions result in both an increase in low‐density growth rate and an increase in equilibrium density in the presence of mutualists.

These studies using the interspecific density dependence approach included more ecological justification for mechanisms that limited benefit accrual. However, phenomenological accounts of environmental conditions limiting population growth were still present with most models via an undiscussed intraspecific limitation term (see discussion by Johnson & Amarasekare, 2013). That is, authors assumed that at least one partner was limited by negative density dependence to ensure curved nullclines and stable coexistence in the mutualism (see *Intraspecific density*‐*dependence*, below). Recently, Moore et al. (2018) introduced nonlinearities in intraspecific limitation while maintaining linear benefits (Table 3, Case 1.1.2‐3). Mutualism is stable when density dependence accelerates with increasing recipient density. Ecologically, this means that the growth rate of the population receiving the benefit decreases faster and faster at higher density, which has been observed empirically (Moore et al., 2018). This result highlights the importance of investigating the effect of more realism in intraspecific limitation on the dynamics of mutualism, which has been largely underexplored.

Other authors derived models with benefits limited by both inter‐ and intraspecific density dependence (Fishman & Hadany, 2010; Johnson & Amarasekare, 2013; May, 1976, 1978; Wells, 1983; Table 3). This added complexity was usually justified by system‐specific considerations (e.g., May, 1976; Wells, 1983), but it also emerges from individual‐level mechanisms in plant‐pollinator systems (Fishman & Hadany, 2010) or intraspecific competition for food or services (Johnson & Amarasekare, 2013). In general, these limitations emerge when systems are limited both by availability of service providers (e.g., pollinators) and by the substrates that receive benefit (e.g., flowers to be pollinated, seeds to germinate, or individuals to protect from predators; Hale et al., 2021).

### 2.3 Cost‐benefit models and shifting net effects

Empirical work bloomed in the 1980s, revealing that mutualisms are not only more (omni)present than previously expected but also context‐dependent (Bronstein, 1994; Chamberlain et al., 2014; Herre et al., 1999; Thompson, 1988). That is, the net effect of these interactions often shifts between mutualism and parasitism or competition due to the relative balance of costs and benefits of participating in the interaction (also called “context‐dependency”). Moreover, costs and benefits themselves may be strongly varying across space, time, and other abiotic conditions. Early investigations of this topic used models that could accommodate different types of interactions through smooth transitions in parameter values (Gilpin et al., 1982; Pierce & Young, 1986; Whittaker, 1975). For example, Pierce and Young (1986) did not provide a specific mathematical form but used a geometric argument to investigate the dynamics of an ant‐lycaenid butterfly interaction in which lycaenids may be mutualistic, commensalistic, or parasitic to tending ants.

Neuhauser and Fargione (2004) explored the mutualism‐parasitism continuum using the classical predator–prey (or host–parasite) Lotka–Volterra model with the additional possibility of the parasite benefiting the host (Figure 3a,b). The model includes both benefits and costs, and it was applied to study plant–mycorrhizae interactions across gradients of soil fertility. The authors assumed that mycorrhizal fungi not only increase host–plant equilibrium density (benefits) but also linearly increase plant death rate due to exploitation (costs). This and other cost‐benefit models can exhibit coexistence equilibria that are stable spirals, meaning that the population densities will oscillate toward a fixed point (see *Patterns from Theory*). Zhang (2003) also modified a Lotka–Volterra model to accommodate mutualism but chose the competition instead of the predator–prey version of the model (Figure 3c,d). The modified model assumed that the interaction between species was competitive at high density and mutualistic at low density, modeled phenomenologically as parabolic nullclines. This model can predict competitive exclusion, competitive coexistence where one partner dominates depending on initial density, thresholds in which low density of one partner drives the system to collapse, or “mutualism” according to the criterion that species coexistence stably at higher density than either could have achieved alone. Unfortunately, it is difficult to understand which of the diverse dynamics this model can exhibit are most ecologically relevant because interpretation is not provided for its parameters. A mechanistic derivation that achieves similar dynamics could be useful future work (but also see Gross, 2008 for a similar approach on an explicit resource).

Other models also described different outcomes depending upon relative species' density (Hernandez, 1998; Holland et al., 2002; Tonkyn, 1986; Wang, 2019). In an important advance, Holland et al. (2002) proposed a suite of models in which different net effects result from the difference between increasing benefit functions and linear, saturating, or decreasing cost functions (see Figure 1 of Holland et al., 2002). Their approach balances out different mechanisms that cause net effects of the interaction to shift as the relative densities of the populations change over time.

In seeking to represent the phenomena or mechanisms of shifting interaction outcomes, cost‐benefit models revealed a much more complex set of potential dynamics for mutualism than had been previously reported. Saturating costs bend species' nullcline toward the partner's axis at high partner density, curving it back around toward the origin into a lobe shape (Figure 3c–f). This is because high partner density exerts high saturating costs on the recipient, which may exceed the benefits that can be acquired. Up to five nontrivial equilibria occur when coexistence is feasible. Moreover, separatrices running through saddle points define basins of attraction that lead to extinction or potential single‐species persistence for facultative species. This ensures instability when one population is of substantially higher density than the other due to overexploitation of the rare partner (Figure 4b). These dynamics contrast with the threshold effects (Figure 4a) wherein the low‐density partner benefits from mutualism but cannot provide sufficient reciprocal services. When the low‐density partner becomes even rarer, it experiences an Allee effect, leading to its extinction (Figure 4b). The high‐density partner will also go extinct if it is obligate upon the low‐density partner.

This much more complex set of potential dynamics that emerges from cost‐benefit models exemplifies the criticism of mutualism theory as either too system‐specific or too abstract to provide general insight into patterns and processes in mutualism (Bronstein, 2001a; Holland, 2015). Additionally, the field had not clearly connected the costs and benefits observed for individuals participating in a mutualism to potential population‐level effects. The time was ripe for a conceptual synthesis.

### 2.4 Consumer‐resource approach to mutualistic interactions

In a landmark work, Holland and DeAngelis (2010) formalized a consumer‐resource approach to mutualism, providing a bridge between mutualism and the ecology of other interspecific interactions. In their framework, mutualisms may be “unidirectional” or “bidirectional” consumer–resource interactions, in which one or both partners benefit from consuming costly resources provided by the other (Figure 4b, Figure 3e,f, respectively). Such framework accommodated the shifting net effects of previous models (Holland & DeAngelis, 2009, previous section), and formalized the concept of ecological costs and benefits as modifications to demographic rates due to resource provisioning and nutrient or service consumption. Notably, this framework allowed mutualisms to be modeled as a dynamic continuum along a spectrum of other interspecific interactions, such as predator–prey and competitive interactions (Holland, 2015; Holland & DeAngelis, 2009). This was possible by clarifying the “currency” of the effects of mutualism as energy or biomass exchanges that manifest in changes to per‐capita growth rate (or its components: birth, death, immigration, etc.). This framework stimulated recent development of theory for more specific systems (e.g., Kang et al., 2011; Martignoni et al., 2020).

Holland and DeAngelis (2010) modeled specific study cases similarly to previous studies (see *Saturating benefits*, above), but with costs defined separately from benefits via saturating interspecific functions, accrued through provisioning resources. In contrast, service‐provisioning by consumers is assumed to incur only fixed costs that can be accounted for in parameter values, like increased handling time when foraging for resources. The nonlinear costs cause lobe‐shaped nullclines allowing up to five coexistence equilibria. Like the earlier Zhang (2003) model, many dynamics are possible including mutualistic stable coexistence and oscillations. However, instead of the competitive exclusion and competitive coexistence outcomes of Zhang's model, “parasitism” by one partner is due to exploitation by a high‐density partner that outweighs the benefits it provides to the lower density partner. In most dynamics of the Holland and DeAngelis model, parasitism collapses the system to extinction instead of allowing a stable but exploitative interaction like in Zhang's model.

Following Holland and DeAngelis' publication, authors began to investigate accounting for resource dynamics in consumer‐resource mutualisms more mechanistically. Resource dynamics were also considered in some earlier literature investigating mutualistic exchange of resources and between guild members sharing resources (bidirectional consumer resource), largely in the context of investigating coexistence mechanisms (e.g., Gross, 2008; McGill, 2005; Meyer et al., 1975). However, Benadi et al. (2012) and Valdovinos et al. (2013) proposed consumer‐resource models for pollination networks (unidirectional consumer resource) in which consumption was on nectar “rewards” rather than individuals of the resource populations directly (but also see Scheuring, 1992 for a similar stage‐structured model). These models separated the dynamics of the plants' vegetative biomass from the dynamics of the plants' floral rewards either implicitly (Benadi et al., 2012, 2013a) or explicitly (Valdovinos et al., 2013). Explicitly separating vegetative and rewards dynamics introduces complexity but allows (1) tracking of the depletion of floral rewards by pollinator consumption, (2) evaluating exploitative competition among pollinator species consuming the floral rewards provided by the same plant species, and (3) incorporating the capability of pollinators to behaviorally increase their foraging effort on the plant species in their diet with more floral rewards available (adaptive foraging). Though these models were developed for plant–pollinator networks, their ideas paved the way for new investigations of mutualism at the pairwise (Hale et al., 2021; Revilla, 2015; Wang, 2019) and community (Benadi et al., 2013b; Hale et al., 2020; Valdovinos et al., 2016) scales. For example, Revilla (2015) assumed rewards achieve steady state compared to changes in population density and derived models in which the linear consumption rate on rewards mediates benefits to the resource species. Hale et al. (2020) considered that pollinator visits can be approximated by consumption of floral rewards, and assumed that benefit to both plant and pollinator species is proportional to consumption rates on floral rewards. Hale et al. (2021) further specified whether benefits should be proportional to per‐capita consumption rate (as may be the case for animal‐dispersed plants) or to total consumption rate (as may be the case for animal‐pollinated plants, which require obligate outcrossing). The latter leads to emergent Allee effects (Courchamp et al., 2018) for obligately animal‐pollinated plants, explained by the plants' inability to attract pollinators at low density.

## 3 PATTERNS FROM THEORY

Historically, theory in mutualism has been focused on understanding how mutualisms can stably persist. Here, we broaden our scope to ask, what dynamics does the theory predict mutualisms will exhibit, and are they dependent upon ecological system or model formulation? We found that predictions for the population dynamics of mutualisms are qualitatively robust across the models reviewed, despite differences in level of detail, types of benefit, and inspiring systems. We synthesize these general findings below.

### 3.1 Mutualisms are stable with intraspecific density dependence and saturating benefits

The stability of mutualistic interactions has been discussed in the community ecology literature for decades (Allesina & Tang, 2012; Bascompte et al., 2006; Hale et al., 2020; Holland, 2015; Holland & DeAngelis, 2010; Johnson & Amarasekare, 2013; May, 1972, 1973; Valdovinos, 2019). Discussion has included definitions of stability (e.g., lack of positive feedbacks, robustness to perturbations), the scale at which they are assessed (e.g., pairwise interactions, between guilds, within communities), and stabilizing mechanisms (e.g., nonrandom interactions, environmental limits, consumer‐resource dynamics).

We found that theoretical investigation of pairwise mutualism has repeatedly and robustly shown that mutualisms are stable. Minimal realism in terms of limited benefits, accumulating costs, or accelerating intraspecific competition allow stable coexistence at high density according to the criteria of local stability analysis. That is, these systems will return to equilibrium after small perturbations to population densities. Under other definitions of stability, such as persistence of populations or return time to equilibrium, mutualisms can be even more stable than predation and competition (Addicott, 1981; Wolin & Lawlor, 1984). Moreover, other mechanisms not reviewed here including spatial structure (Amarasekare, 2004; Armstrong, 1987; Mohammed et al., 2018), rewards or resource dynamics (Cropp & Norbury, 2019; Gross, 2008; Meyer et al., 1975; Revilla, 2015; Scheuring, 1992; Wang, 2019), adaptive foraging (Valdovinos et al., 2013, 2016, 2018), and predators or competitors (Addicott & Freedman, 1984; Hale et al., 2020; Heithaus et al., 1980; Mougi & Kondoh, 2012; Rai et al., 1983; Ringel et al., 1996; Tonkyn, 1986) also stabilize mutualisms.

The pattern of stable coexistence of mutualists at high density is robust across mechanisms that limit benefit (Figures 2 and 3, Table 3). Both inter‐ and intraspecific density dependence in saturating benefit functions lead to the same qualitative dynamics when they are present in at least one partner (also see *Thresholds*, below). However, intraspecific density dependence and its effect on stability have been a source of confusion in the mutualism literature for decades.

#### 3.1.1 Intraspecific density dependence

We found that authors described their models as exhibiting intraspecific density dependence in three (not necessarily distinct) cases. In the first case, authors are referring to the negative density dependence term in a simple population dynamic model (Case 1 of Table 3). This term causes the decline in per‐capita growth rate with increasing population density, and historically was modeled through a carrying capacity function ($‐N_{i}/K_{i}$ in Table 3). It is now typically modeled through a “self‐limitation” term ($‐s_{i}N_{i}$ in Table 3), though it may represent any form of negative density dependence such as the Janzen‐Connell effect, not just intraspecific competition for limited resources. To display a nullcline in the relevant ecological quadrant, it is necessary for mutualism models to include nonzero negative density dependence unless they include some other source of dependence on recipient density (e.g., $s_{i}$ can be zero in Case 2 of Table 3 because benefit saturates in terms of recipient density). Moore et al. (2018) found that one species having an accelerating negative density dependence term is also sufficient to allow stable coexistence if per‐capita benefits accrue linearly (Case 1.1.2). However, the form of negative density dependence (accelerating, decelerating, or constant) does not typically affect nullcline geometry if per‐capita benefits saturate (e.g., does not affect the qualitative dynamics of Cases 1.2, 2.1, 2.2 of Table 3).

In the second case, authors refer to intraspecific density dependence in their models when benefits from mutualism increase per‐capita growth rate directly (i.e., affect density‐independent rates such as increased per‐capita birth rate or decreased per‐capita death rate), but benefits saturate with increasing recipient density (Case 2 of Table 3). This emerges when benefits are a function of the partner's visitation rate on the recipient or consumption rate on rewards provided by the recipient or when the recipient has limited substrate with which to convert interactions into benefits. This may generally be the case when mutualists provide reproductive or protective services (e.g., Soberón & Martinez del Rio, 1981, Thompson et al., 2006, Johnson & Amarasekare, 2013, Hale et al., 2021, but also see nutritional exchanges in Parker, 2001, Martignoni et al., 2020).

In the third case, authors refer to intraspecific density dependence when benefits from mutualism reduce negative density dependence (Case 3 of Table 3) so that the effect of mutualism is most prominent at high recipient density (Wolin & Lawlor, 1984). Authors have chosen this approach when mutualists provision habitat (e.g., Thompson et al., 2006), reduce density‐dependent mortality such as seed predation via the Janzen‐Connell effect (e.g., Hale et al., 2021), or in the case of symbionts, which live within host populations (e.g., Whittaker, 1975). Here, benefits may be mediated through carrying capacity (Case 3.1) or through a self‐limitation term (Cases 3.2, 3.3), with different resulting nullcline geometries. Linear increases in carrying capacity or decreases in self‐limitation rate can yield unbounded population growth (Table 3). More generally, even models with saturating benefits can exhibit unstable behavior when benefits accrue directly to a term that represents intraspecific density dependence, which decreases per‐capita growth rate at high density (not shown). If mutualism decreases negative density dependence to such an extent that it induces positive density dependence at high partner density, the recipient population will begin accruing increasing benefit with its own increasing density (Case 3.2). Then, the system can display unbounded growth (Figure 2c,d) unless benefits are additionally limited by extrinsic or intrinsic factors such as the number of seeds that can germinate after seed dispersal or the number of ovules that can be pollinated by pollinators (Case 3.3, Figure 3h).

Though all three of the above cases have been called “intraspecific density‐dependence” in the mutualism literature, they refer to different ecological phenomena and have different implications for the dynamics of mutualism. All models must include some form of per‐capita dependence on recipient density for feasible nullclines, but this may be manifest through a self‐limitation term or through per‐capita benefit functions that decrease with increasing recipient density. Models in which benefits reduce negative density dependence in a recipient population tend to allow unbounded population growth unless there are additional limits to benefits accrued. In contrast, models in which per‐capita benefits saturate with increasing recipient density are stable, and exhibit the robust dynamics of high density stable coexistence and a low‐density threshold observed in models with benefits that saturate with increasing partner density (i.e., interspecific density dependence).

### 3.2 Mutualisms exhibit thresholds when at least one partner is obligate

Nearly all models that predict stable coexistence at high density also predict destabilizing thresholds at low density when one or more partners are obligate upon the mutualism (Figure 2a,b,e,f, Figure 3a,b,g,h). Specifically, if either species dips below a critical threshold in population density, the obligate partner(s) will go extinct, even if initially at high density (Figure 4a). This collapse occurs because, under the threshold, the low‐density species cannot provide sufficient benefits to its higher density partner. Threshold effects occur in systems with interaction strengths high enough to allow feasible coexistence, but with per‐capita growth rates small enough (very negative for obligate partners, near‐zero for facultative partners) that a partner can potentially achieve densities low enough for long enough that its obligate partner will go extinct.

Understanding threshold dynamics provides rich insight into interaction strength, obligacy, and positive feedbacks in mutualistic interaction. By definition, obligate mutualists have negative per‐capita growth rate in the absence of their partner. Thus, obligate mutualists can only be saved from population decline by benefits from mutualism that exceed their own negative intrinsic growth rate, that is, via strong mutualistic interactions. If both partners are initially at high enough density, obligate mutualists can achieve positive population growth, resulting in stable coexistence. However, if an obligate mutualist is at high density but its partner is at low density, the obligate mutualist will decline quickly due to both its negative intrinsic growth rate and strong intraspecific limitation at high density. The low‐density partner may be growing due to mutualistic benefits, positive intrinsic growth, or release from intraspecific limitation. However, under the threshold, its population cannot recover fast enough to provide sufficient benefit to cancel out the negative intrinsic growth rate of the obligate partner and save it from decline. On the other hand, facultative partners can rely upon their own positive intrinsic growth rate to recover from low density, even after declines due to strong intraspecific competition or insufficient benefits provided by its partner. Thus, destabilizing threshold effects do not occur when both partners are facultative. However, highly nonlinear models can exhibit similar thresholds in facultative partnerships where coexistence occurs below the threshold at low, rather than high densities (“bistable coexistence,” Hale et al., 2021; Parker, 2001).

Threshold dynamics emerge from the unique nature of mutualism and are potentially characteristic of this interaction. In predator–prey interactions, a low‐density predator may benefit from a higher density prey population that is declining, but negative feedback in the system also limits the growth of the predator population at high density and subsequently allows the recovery of the prey population from low density. In competition interactions, the higher density partner exerts stronger and stronger negative effects on the rare population, causing the rarer population to go extinct if interspecific competition exceeds intraspecific competition for at least one of the competitors. In contrast, the positive feedback in the mutualistic system requires that both partners can provide sufficient benefits to the other to maintain the interaction. Notably, threshold effects also occur in models that take very different approaches than those reviewed here. For example, Ingvarsson and Lundberg (1995) observed threshold effects dependent upon the ability for pollinators to find flowers in a modified disease model for mutualism, while Wang (2019) showed that the thresholds observed in Revilla's (2015) model more precisely occur between pollinator and rewards density rather than pollinator and plant density directly. This further emphasizes the potential generality of thresholds in mutualisms.

#### 3.2.1 Allee effects

Allee effects are a form of threshold where the population exhibits negative per‐capita growth rate when rare. Here, we use “Allee effects” to refer specifically to strong, demographic Allee effects (Kramer et al., 2009) that emerge from the mutualism (i.e., are not hard coded into the population dynamics, Courchamp et al., 2018). Allee effects can emerge from many mechanisms, but we distinguish between a few proximal causes that suggest differing management recommendations for driving a collapsing system to high‐density stable coexistence. The most obvious case is also the least common form of threshold observed in mutualism models: Allee effects driven by the inability of a population to support itself. This type of Allee effect has also been observed in food chains that include protection mutualism (Morales et al., 2008) and in models of sequential colonization of patches by plants and mobile mutualists (Amarasekare, 2004). As mentioned above, Hale et al. (2021) find Allee effects in obligate plants when they become too rare to attract sufficient visitation from pollinators (Figure 4c). From a management perspective, it would be necessary to supplement the population experiencing the Allee effect (the declining, low‐density partner) to prevent its extinction (Figure 4c). The partner‐induced threshold described above also leads to Allee effects, wherein species decline when their partner is too low in density to support positive growth. In this case, it would also be necessary to supplement the low‐density species, though it may already appear to be recovering due to positive population growth and high partner density. Indeed, from a management perspective, this would achieve the counter‐intuitive goal not of saving the low‐density population, but rather its high‐density partner from extinction (Figure 4a). Finally, Holland and DeAngelis (2010) find Allee effects in animal populations induced by overexploitation from another consumer mutualist. In this case, the management recommendation would be to equalize partners' population densities to avoid overexploitation (Figure 4b).

### 3.3 Strong interactions are needed for obligate mutualists to persist

Research on mutualistic interactions has yet to firmly define interaction strength (Valdovinos, 2019). In Lotka–Volterra models, interaction strength is simply defined by the benefit coefficient (*α_ij_* in Equations 1, 2, 4). However, as authors have gained deeper mechanistic understanding of mutualism, it has become clear that interaction strength is a more complex topic related to the “effectiveness” of mutualistic partners (Schupp et al., 2017; Vázquez et al., 2015). Schupp et al. defined the effectiveness of a population for providing mutualistic benefits to its partner as the product of the “quantity” and “quality” of benefits provided. The term “quality” accounts for the species‐specific and interaction‐specific traits, as well as the environmental context that determine how much benefit a partner can receive from a unit of benefit “quantity”. Examples of such benefit quality are the nutrition acquired from a foraging visit or the probability of a seed recruiting after being removed by a disperser.

The parameters that determine the quality of the mutualistic interaction are useful for understanding the criteria for stable coexistence and thresholds. Weak interactions between facultative partners in Lotka–Volterra models are considered stabilizing because they ensure stable coexistence instead of permitting unbounded growth. Specifically, mutual benefits must be weaker than species' intraspecific limitation (Gause & Witt, 1935; Travis & Post, 1979). However, stable coexistence always occurs between facultative mutualists in models with saturating nullclines regardless of interaction strength. Conversely, in saturating systems with at least one obligate partner, interactions must be sufficiently strong to overcome the negative intrinsic growth rate of the obligate partner for coexistence to be feasible (Bazykin, 1997). In this case, destabilizing threshold effects can occur not because of interaction strength, but due to the low intrinsic growth rate of the partner. Overall, stronger interactions stabilize systems with threshold effects by decreasing the threshold in population density that causes the system to collapse, which allows positive growth from lower densities.

### 3.4 Effects of mutualism varies between low and high population density

Empirical work has shown that the effects of mutualism vary with both recipient (Wolin & Lawlor, 1984) and partner density (Holland, 2015), and models show that this can lead to different ecological dynamics. When benefits are strongest at low recipient density, we can expect the robust dynamics of stable coexistence and threshold effects described previously (Figure 2). When benefits are strongest at high recipient density, models predict unbounded growth unless limited by other intrinsic or extrinsic factors (compare Figure 2c,d to Figure 3h). When benefits are strongest at intermediate recipient density, we can expect saturating dynamics and emergent Allee effects (Figure 4b). On the other hand, if benefits are strongest at low partner density and turn into net costs at high partner density, two outcomes are possible (Figure 3, Figure 4c): competitive or exploitative dynamics if the partner is at too high of an initial density, or potential oscillations to stable coexistence if the partners are well‐balanced.

Early syntheses reported that mutualism with the strongest effects at high recipient density is less likely to be stable than that with the strongest effects at low recipient density (Addicott, 1981; Wolin, 1985). At that time, authors represented high‐density effects of mutualism as direct modifications to species' carrying capacity (Equations 2, S9, S16; Wolin & Lawlor, 1984). Authors now represent the effects of mutualism exclusively through changes in demographic rates (Holland, 2015) unless explicitly representing habitat provisioning, for example, corals or plants with domatia and their animal partners (Thompson et al., 2006). Mutualism may still have the strongest effects at high density (e.g., if benefits reduce negative density dependence due to intraspecific competition or the Janzen‐Connell effect), but this would be represented by modifying intraspecific limitation due to mutualism. Categorizing mutualisms by their relative magnitude of costs and benefits at low versus high density of recipients versus partners is still a profitable approach that could lead to a next‐generation theoretical framework that organizes mutualism by their population dynamics. Additionally, separating out the specific demographic rates affected by mutualistic interactions (as in Thompson et al., 2006 and Hale et al., 2021) will likely clarify the differences and similarities between mutualisms. Even if the population dynamics of most models of mutualisms are qualitatively robust, the details of the low‐density dynamics and the criteria for collapse can provide insight for system‐specific mechanisms and patterns among them (Hale et al., 2021; Wu et al., 2019).

### 3.5 Costs of mutualism can cause damped and undamped oscillations

Models that incorporate costs to the mutualistic interaction can exhibit the same qualitative dynamics described above. That is, they are stable when incorporating limiting factors to benefits and self‐limitation, exhibit thresholds when at least one partner is obligate, and need strong interactions for obligate partners to persist. Additionally, these models can produce oscillations. Linear costs can result in damped oscillations when the equilibrium is a stable spiral (Figure 3b,g; Kang et al., 2011; Neuhauser & Fargione, 2004). Nonlinear costs can cause undamped oscillations when the equilibrium is a stable center (Figure 3d,f; Holland & DeAngelis, 2010; Zhang, 2003).

Undamped oscillations occur when overexploitation by the consumer causes an Allee effect in the resource, which does not necessarily lead to extinction (Figure 3f). After depleting their resource population, the consumer population also declines, eventually allowing the resource to receive sufficient benefit compared to losses due to consumption. The system thus recovers, and coexistence is maintained in this region via a limit cycle (i.e., oscillations) around a stable center (left‐most stable equilibrium, Figure 3f). This outcome is not seen in simpler models without cost terms, which predict stable coexistence at a nonoscillatory node (Figure 2), or with linear cost terms, which can predict damped oscillations in a stable spiral (Figure 3b,g).

Note that oscillation has been considered an important dynamic for mutualism models to reiterate, as justified by observations that mutualist populations can vary in space and time (Holland, 2015). However, such variability need not necessarily be driven by the underlying population dynamics. Far simpler models of mutualism can produce oscillations when accounting for discrete time dynamics (e.g., Gilpin et al., 1982). Additionally, population oscillations observed in nature may be caused by external factors, such as environmental variation. This emphasizes that introducing explicit cost terms into mutualism should be adequately justified at the population level. Regardless, the models in question suggest that oscillations can be induced predictably, for example, by decreasing the density‐dependent mortality of an obligate symbiont (Neuhauser & Fargione, 2004, e in Equation. 9), which could potentially be tested empirically by using different fungal strains in a plant‐mycorrhizal system (Martignoni et al., 2021).

## 4 DISCUSSION

Theoretical study of mutualism has lagged behind the other two “pillars” of community ecology: competition and predator–prey interactions (Callaway, 2007; Holland, 2015). Early theory of mutualistic interactions was contemporaneous with early theory on predator–prey and competition interactions. After a gap of nearly 40 years, the destabilizing influence of mutualistic interactions in communities reignited theoretical attention. More recently, theory of mutualistic networks has made faster progress than that of pairwise mutualisms (Bascompte et al., 2003, 2006; Benadi et al., 2013b; Hale et al., 2020; Holland et al., 2006; Okuyama & Holland, 2008; Thébault & Fontaine, 2010; Valdovinos, 2019; Valdovinos et al., 2018, 2013, 2016), and has also garnered more attention from broader community ecology (e.g., McCann & Gellner, 2020).

Ecological theory of mutualism has been criticized as sparse, largely consisting of models that are either too abstract to be useful or too case‐specific to reveal general patterns (Bronstein, 2015a). This is an accurate description of many of the models we reviewed; however, remarkably, nearly all these models conformed to the same dynamics. We found that many historical models make similar qualitative predictions despite their different derivations, mechanisms, and inspiring systems. When feasible, coexistence is stable, and populations grow with bound. Mutualisms with at least one obligate partner exhibit thresholds, under which the low density of one partner destabilizes the system. If a species sustains nonlinear, population‐level costs from mutualism, it may be overexploited to extinction by its partner. These patterns suggest that there exists a robust population dynamic theory of mutualism that can make general predictions. With this groundwork of theory laid, authors can now focus on how relaxing the assumptions of current models affects their predictions. For example, spatial and transmission models reiterate the threshold predictions of models that conform to the mean‐field assumption (Ingvarsson & Lundberg, 1995; Mohammed et al., 2018) as do models with explicit rewards dynamics compared to those that approximate rewards dynamics as at steady‐state (Revilla, 2015; Wang, 2019).

### 4.1 Avenues for future research

Future work should understand how predictions from pairwise models scale to the network level. Threshold effects only occur when at least one partner is an obligate mutualist. Most species have multiple potential partners and thus are not truly “obligate” in the sense that only a specific pairwise interaction can allow positive population growth. Instead, most mutualists are likely to be facultative, engaging in diffuse interactions with many potential partners. However, it is likely that mortality exceeds reproduction in the absence of mutualistic interactions for many species. In this sense, species may be obligate mutualists even though they have multiple partners. Additionally, species are likely to have critical (cumulative) thresholds to allow population growth. For example, Valdovinos and Marsland (2021) identify the quality of visits needed from pollinators for plants to persist. Below such threshold, the plant species and the animals depending on those plants go extinct. Understanding how destabilizing thresholds may emerge or be ameliorated due to obligate mutualists in a network setting is an important goal for future work. Moreover, emphasis on consumer‐resource approaches with a common “currency” of energy or biomass flows (Holland, 2015) makes mutualisms amenable to integration into interspecific network models such as food webs (e.g., Hale et al., 2020). Such integration can illuminate how context mediates interaction outcomes between potential mutualists, for example by shifting interactions into overexploitation or competition regimes. Indeed, understanding the structure and dynamics of these “multiplex” ecological networks that include multiple types of interactions has been identified as a primary goal in ecology (Kéfi et al., 2012).

Future work should also interrogate the assumptions and predictions of these models empirically. A main assumption is that mutualisms have population‐level impacts. However, most empirical studies quantify the benefits and costs of mutualisms at the individual level in terms of fitness or even by using a single proxy for fitness (Bronstein, 2001a; Ford et al., 2015). Those effects do not necessarily imply population‐ and community‐level impacts of mutualism (Flatt & Weisser, 2000; Ford et al., 2015; Palmer et al., 2010; Williamson, 1972). Therefore, empirical work is of foremost importance to evaluate whether mutualisms affect the population dynamics of mutualistic partners. Among the predictions of these models (stable coexistence, threshold effects, overexploitation), threshold effects have received the most attention (Latty & Dakos, 2019), but more empirical work is still needed. Wotton and Kelly (2011) and Kang et al. (2011) observed threshold effects directly in frugivory systems and in ant‐fungal gardens, respectively, although the authors did not identify their results as such. Hale et al. (2021) showed that threshold effects in obligate plants may be swamped out by Allee effects (e.g., Forsyth, 2003), which suggests that targeted experiments to explore population trajectories should consider the criteria for observing different dynamics (Figure 4).

One difficulty of empirical applications is that an out‐of‐the‐box consumer‐resource approach following Holland and DeAngelis' (2010) framework can be logistically overwhelming. Nonlinear cost and benefit functions generate so many dynamics that they are nearly intractable analytically (but see numerical toolkit by Wu et al., 2019). Moreover, with up to four separate functional responses to parameterize, this framework requires an extremely high number of parameters to estimate empirically. This level of detail may be necessary to describe some two‐species mutualism but is likely not general. Simplifications like approximating costs and benefits as proportional to consumers' foraging rate (Hale et al., 2021; Revilla, 2015; Soberón & Martinez del Rio, 1981) can facilitate integration between theoretical and empirical approaches. Additionally, costs that scale with rewards construction can be approximated as fixed reductions to benefit, and thus accounted for in the measured parameters (Hale et al., 2021; Revilla, 2015; Figure 3h). Systems with these complementary saturating benefits and fixed costs are likely to display much more limited dynamics than those shown in Figure 3c–f. For example, Kang et al. (2011) and Martignoni et al. (2020), Martignoni et al. (2021) adapted Holland and DeAngelis' approach to specific empirical systems, leading to models that predict the threshold and stable coexistence dynamics of simpler saturating benefit models (Figure 3g).

Reviewers for an earlier version of this manuscript commented that our results cement the idea that pairwise models of mutualism have been “pushed…as far as they will go,” that “this literature has limited usefulness for motivating the theory of the future,” and that it may be “the nature of mutualism” that its dynamics are “not very interesting…for a broad audience in ecology and evolution.” Though we cannot speak to whether mutualism is of interest to specific individuals, we do believe that this attitude may have contributed to the long‐term stagnation and repeated loss and rediscovery of theory in mutualism. A clear summary of the population dynamics of pairwise mutualisms (as we presented here) is an important groundwork for directing research into modules and networks including mutualistic interactions, the evolutionary origins of mutualism, and, pressingly, directing conservation efforts across systems (Figure 4). Both within the discipline and more broadly, there is an impression that theory is lacking. But it is simply not the case that ecological theory of mutualism is incoherent or underdeveloped: we find here that it is remarkably self‐consistent despite the diversity of inspiring systems and modeling frameworks. It is not a mystery how pairwise mutualisms can persist stably, at least theoretically. Mutualisms are highly stable at high density, and the network setting may diffuse the risk of low density‐thresholds leading to population collapse. A similar set of empirical literature to support or dispute the models' results has yet to accumulate, but we hope that by clearly outlining dynamical expectations of mutualistic theory, such work will be more accessible to empiricists.

## CONFLICT OF INTEREST

The authors declare no competing interests.

## AUTHOR CONTRIBUTIONS

**Kayla R. S. Hale:** Formal analysis (lead); methodology (equal); software (lead); validation (lead); visualization (lead); writing – original draft (lead); writing – review and editing (equal). **Fernanda S. Valdovinos:** Conceptualization (lead); methodology (equal); resources (lead); supervision (lead); writing – review and editing (equal).

## Supporting information

Table S1

## Data Availability

No new data were used in this work. Mathematica notebooks used to analyze models and generate figures are available at https://doi.org/10.5061/dryad.0p2ngf230.

## References

[ece38453-bib-0001] Addicott, J. F. (1979). A multispecies aphid‐ant association: Density dependence and species‐specific effects. Canadian Journal of Zoology, 57, 558–569. 10.1139/z79-066

[ece38453-bib-0002] Addicott, J. F. (1981). Stability properties of 2‐species models of mutualism: Simulation studies. Oecologia, 49, 42–49. 10.1007/BF00376896 28309447

[ece38453-bib-0003] Addicott, J. F. , & Freedman, H. I. (1984). On the structure and stability of mutualistic systems: Analysis of predator‐prey and competition models as modified by the action of a slow‐growing mutualist. Theoretical Population Biology, 26, 320–339. 10.1016/0040-5809(84)90037-6

[ece38453-bib-0004] Albrecht, F. , Gatzke, H. , Haddad, A. , & Wax, N. (1974). The dynamics of two interacting populations. Journal of Mathematical Analysis and Applications, 46, 658–670. 10.1016/0022-247X(74)90267-4

[ece38453-bib-0005] Allesina, S. , & Tang, S. (2012). Stability criteria for complex ecosystems. Nature, 483, 205–208. 10.1038/nature10832 22343894

[ece38453-bib-0006] Amarasekare, P. (2004). Spatial dynamics of mutualistic interactions. Journal of Animal Ecology, 73, 128–142. 10.1046/j.0021-8790.2004.00788.x

[ece38453-bib-0007] Armstrong, R. A. (1987). A patch model of mutualism. Journal of Theoretical Biology, 125, 243–246. 10.1016/S0022-5193(87)80045-0

[ece38453-bib-0008] Bascompte, J. , & Ferrera, A. (2020). A structural theory of mutualistic networks. In K. S. McCann , & G. Gellner (Eds.), Theoretical ecology. Oxford University Press.

[ece38453-bib-0009] Bascompte, J. , Jordano, P. , Melián, C. J. , & Olesen, J. M. (2003). The nested assembly of plant‐animal mutualistic networks. Ecology, 100, 9383–9387. 10.1073/pnas.1633576100 PMC170927 12881488

[ece38453-bib-0010] Bascompte, J. , Jordano, P. , & Olesen, J. M. (2006). Asymmetric coevolutionary networks facilitate biodiversity maintenance. Science, 312, 3–5. 10.1126/science.1123412 16627742

[ece38453-bib-0011] Bazykin, A. D. (1997). In A. I. Khibnik , & B. Krauskopf (Eds.), Competition and symbiosis. In: Nonlinear dynamics of interacting populations (pp. 101–116). World Scientific Publishing.

[ece38453-bib-0012] Benadi, G. , Blüthgen, N. , Hovestadt, T. , & Poethke, H. J. (2013a). Contrasting specialization‐stability relationships in plant‐animal mutualistic systems. Ecological Modelling, 258, 65–73. 10.1016/j.ecolmodel.2013.03.002

[ece38453-bib-0013] Benadi, G. , Blüthgen, N. , Hovestadt, T. , & Poethke, H. J. (2013b). When can plant‐pollinator interactions promote plant diversity? American Naturalist, 182, 131–146. 10.1086/670942 23852349

[ece38453-bib-0014] Benadi, G. , Blüthgen, N. , Hovestadt, T. , Poethke, H. J. , Day, T. , & Shaw, R. G. (2012). Population dynamics of plant and pollinator communities: Stability reconsidered. American Naturalist, 179, 157–168. 10.1086/663685 22218306

[ece38453-bib-0015] Boit, A. , Martinez, N. D. , Williams, R. J. , & Gaedke, U. (2012). Mechanistic theory and modelling of complex food‐web dynamics in Lake Constance. Ecology Letters, 15, 594–602. 10.1111/j.1461-0248.2012.01777.x 22513046

[ece38453-bib-0016] Boucher, D. H. (1985). The idea of mutualism, past and present. In D. H. Boucher (Ed.), The biology of mutualism (pp. 1–28). Oxford University Press.

[ece38453-bib-0017] Bronstein, J. L. (1994). Conditional outcomes in mutualistic interactions. Trends in Ecology & Evolution, 9, 214–217. 10.1016/0169-5347(94)90246-1 21236825

[ece38453-bib-0018] Bronstein, J. L. (2001a). The costs of mutualism. American Zoologist, 41, 825–839. 10.1093/icb/41.4.825

[ece38453-bib-0019] Bronstein, J. L. (2001b). The exploitation of mutualisms. Ecology Letters, 4, 277–287. 10.1046/j.1461-0248.2001.00218.x

[ece38453-bib-0020] Bronstein, J. L. (2015a). Introduction to Section 1. In J. L. Bronstein (Ed.), Mutualism (pp. 1–2). Oxford University Press.

[ece38453-bib-0021] Bronstein, J. L. (2015b). The study of mutualism. In J. L. Bronstein (Ed.), Mutualism (pp. 3–19). Oxford University Press.

[ece38453-bib-0022] Callaway, R. M. (2007). Positive interactions and interdependence in plant communities (1st ed.) Springer.

[ece38453-bib-0023] Chamberlain, S. A. , Bronstein, J. L. , & Rudgers, J. A. (2014). How context dependent are species interactions? Ecology Letters, 17, 881–890. 10.1111/ele.12279 24735225

[ece38453-bib-0024] Christiansen, F. B. , & Fenchel, T. M. (1977). Theories of populations in biological communities. Springer‐Verlag.

[ece38453-bib-0025] Courchamp, F. , Berec, L. , Gascoigne, J. et al (2018). Population dynamics: Modeling demographic Allee effects. In F. Courchamp (Ed.), Allee effects in ecology and evolution (pp. 63–130). Oxford University Press.

[ece38453-bib-0026] Cropp, R. , & Norbury, J. (2019). Resource‐based models of mutualism. Environmental Modeling and Assessment, 24, 405–420. 10.1007/s10666-018-9646-y

[ece38453-bib-0027] Dean, A. M. (1983). A simple model of mutualism. The American Naturalist, 121, 409–417. 10.1086/284069

[ece38453-bib-0028] Douglas, A. E. (2015). The special case of symbioses: mutualisms with persistent contact. In J. L. Bronstein (Ed.), Mutualism, 20–34. Oxford University Press.

[ece38453-bib-0029] Fishman, M. A. , & Hadany, L. (2010). Plant‐pollinator population dynamics. Theoretical Population Biology, 78, 270–277. 10.1016/j.tpb.2010.08.002 20736029

[ece38453-bib-0030] Flatt, T. , & Weisser, W. W. (2000). The effects of mutualistic ants on aphid life. Ecology, 81, 3522–3529.

[ece38453-bib-0031] Ford, K. R. , Ness, J. H. , Bronstein, J. L. , & Morris, W. F. (2015). The demographic consequences of mutualism: Ants increase host‐plant fruit production but not population growth. Oecologia, 179, 435–446. 10.1007/s00442-015-3341-3 26003308

[ece38453-bib-0032] Forsyth, S. A. (2003). Density‐dependent seed set in the Haleakala silversword: Evidence for an allee effect. Oecologia, 136, 551–557. 10.1007/s00442-003-1295-3 12783298

[ece38453-bib-0033] García‐Algarra, J. , Galeano, J. , Pastor, J. M. , Iriondo, J. M. , & Ramasco, J. J. (2014). Rethinking the logistic approach for population dynamics of mutualistic interactions. Journal of Theoretical Biology, 363, 332–343. 10.1016/j.jtbi.2014.08.039 25173080

[ece38453-bib-0034] Gause, G. F. (1934). The struggle for existence. Williams & Williams.

[ece38453-bib-0035] Gause, G. F. , & Witt, A. A. (1935). Behavior of mixed populations and the problem of natural selection. The American Naturalist, 69, 596–609. 10.1086/280628

[ece38453-bib-0036] Gilpin, M. E. , Case, T. J. , & Bender, E. A. (1982). Counterintuitive oscillations in systems of competition and mutualism. The American Naturalist, 119, 584–588. 10.1086/283935

[ece38453-bib-0037] Goh, B. S. (1979). Stability in models of mutualism. American Naturalist, 113, 261–275. 10.1086/283384

[ece38453-bib-0038] Gotelli, N. J. (2008). A primer of ecology (4th ed.). Sinauer Associates Inc.

[ece38453-bib-0039] Graves, W. G. , Peckham, B. , & Pastor, J. (2006). A bifurcation analysis of a differential equations model for mutualism. Bulletin of Mathematical Biology, 68, 1851–1872. 10.1007/s11538-006-9070-3 16937233

[ece38453-bib-0040] Gross, K. (2008). Positive interactions among competitors can produce species‐rich communities. Ecology Letters, 11, 929–936. 10.1111/j.1461-0248.2008.01204.x 18485001

[ece38453-bib-0041] Hale, K. R. S. , Maes, D. P. , & Valdovinos, F. S. (2021). Dynamics of pollination and seed dispersal mutualisms at low density. (In review). 10.1086/720204 35905405

[ece38453-bib-0042] Hale, K. R. S. , Valdovinos, F. S. , & Martinez, N. D. (2020). Mutualism increases diversity, stability, and function of multiplex networks that integrate pollinators into food webs. Nature Communications, 11, 1–14. 10.1038/s41467-020-15688-w PMC7195475 32358490

[ece38453-bib-0043] Hastings, A. , & Gross, L. (Eds.) (2012). Encyclopedia of theoretical ecology. University of California Press.

[ece38453-bib-0044] Heithaus, E. R. , Culver, D. C. , & Beattie, A. J. (1980). Models of some ant‐plant mutualisms. The American Naturalist, 116, 347–361. 10.1086/283632

[ece38453-bib-0045] Hernandez, M. J. (1998). Dynamics of transitions between population interactions: A nonlinear interaction α‐function defined. Proceedings of the Royal Society B‐Biological Sciences, 265, 1433–1440.

[ece38453-bib-0046] Herre, E. A. , Knowlton, N. , Mueller, U. G. , & Rehner, S. A. (1999). The evolution of mutualisms: Exploring the paths between conflict and cooperation. Trends in Ecology & Evolution, 14, 49–53. 10.1016/S0169-5347(98)01529-8 10234251

[ece38453-bib-0047] Hoeksema, J. D. , & Bruna, E. M. (2000). Pursuing the big questions about interspecific mutualism: A review of theoretical approaches. Oecologia, 125, 321–330. 10.1007/s004420000496 28547326

[ece38453-bib-0048] Holland, J. N. (2015). Population ecology of mutualism. In J. L. Bronstein (Ed.), Mutualism (pp. 133–158). Oxford University Press.

[ece38453-bib-0049] Holland, J. N. , & DeAngelis, D. L. (2009). Consumer‐resource theory predicts dynamic transitions between outcomes of interspecific interactions. Ecology Letters, 12, 1357–1366. 10.1111/j.1461-0248.2009.01390.x 19807773

[ece38453-bib-0050] Holland, J. N. , & DeAngelis, D. L. (2010). A consumer‐resource approach to the density‐dependent population dynamics of mutualism. Ecology, 91, 1286–1295. 10.1890/09-1163.1 20503862

[ece38453-bib-0051] Holland, J. N. , DeAngelis, D. L. , & Bronstein, J. L. (2002). Population dynamics and mutualism: Functional responses of benefits and costs. American Naturalist, 159, 231–244. 10.1086/338510 18707376

[ece38453-bib-0052] Holland, J. N. , Okuyama, T. , & DeAngelis, D. L. (2006). Comment on “Asymmetric Coevolutionary Networks Facilitate Biodiversity Maintenance”. Science, 313(5795), 1887. 10.1126/science.1129547 17008511

[ece38453-bib-0053] Ingvarsson, P. K. , & Lundberg, S. (1995). Pollinator functional response and plant population dynamics: Pollinators as a limiting resource. Evolutionary Ecology, 9, 421–428. 10.1007/BF01237764

[ece38453-bib-0054] Janzen, D. H. (1985). The natural history of mutualism. In D. H. Boucher (Ed.), The Biology of Mutualism (pp. 40–99). Oxford University Press.

[ece38453-bib-0055] Johnson, C. A. , & Amarasekare, P. (2013). Competition for benefits can promote the persistence of mutualistic interactions. Journal of Theoretical Biology, 328, 54–64. 10.1016/j.jtbi.2013.03.016 23542049

[ece38453-bib-0056] Jones, E. I. , Afkhami, M. E. , Akçay, E. , Bronstein, J. L. , Bshary, R. , Frederickson, M. E. , Heath, K. D. , Hoeksema, J. D. , Ness, J. H. , Pankey, M. S. , Porter, S. S. , Sachs, J. L. , Scharnagl, K. , & Friesen, M. L. (2015). Cheaters must prosper: Reconciling theoretical and empirical perspectives on cheating in mutualism. Ecology Letters, 18, 1270–1284. 10.1111/ele.12507 26388306

[ece38453-bib-0057] Kang, Y. , Clark, R. , Makiyama, M. , & Fewell, J. (2011). Mathematical modeling on obligate mutualism‐ Interactions between leaf‐cutter ants and their fungus garden. Journal of Theoretical Biology, 289, 116–127. 10.1016/j.jtbi.2011.08.027 21903102

[ece38453-bib-0058] Kéfi, S. , Berlow, E. L. , Wieters, E. A. , Navarrete, S. A. , Petchey, O. L. , Wood, S. A. , Boit, A. , Joppa, L. N. , Lafferty, K. D. , Williams, R. J. , Martinez, N. D. , Menge, B. A. , Blanchette, C. A. , Iles, A. C. , & Brose, U. (2012). More than a meal.. integrating non‐feeding interactions into food webs. Ecology Letters, 15, 291–300. 10.1111/j.1461-0248.2011.01732.x 22313549

[ece38453-bib-0059] Klein, A.‐M. , Vaissière, B. E. , Cane, J. H. , Steffan‐Dewenter, I. , Cunningham, S. A. , Kremen, C. , & Tscharntke, T. (2007). Importance of pollinators in changing landscapes for world crops. Proceedings of the Royal Society B: Biological Sciences, 274, 303–313. 10.1098/rspb.2006.3721 PMC1702377 17164193

[ece38453-bib-0060] Kostitzin, V. A. (1934). In G. Teissier (Ed.), Symbiose, parasitismine et evolution (Etude mathématique). Actualités scientifiques et industrielles, Hermann et Cie. https://www.worldcat.org/title/symbiose‐parasitisme‐et‐evolution‐letude‐mathematique/oclc/419232901

[ece38453-bib-0061] Kot, M. (2001). Elements of mathematical ecology. Cambridge University Press.

[ece38453-bib-0062] Kramer, A. M. , Dennis, B. , Liebhold, A. M. , & Drake, J. M. (2009). The evidence for Allee effects. Population Ecology, 51, 341–354. 10.1007/s10144-009-0152-6

[ece38453-bib-0063] Latty, T. , & Dakos, V. (2019). The risk of threshold responses, tipping points, and cascading failures in pollination systems. Biodiversity and Conservation, 28, 3389–3406. 10.1007/s10531-019-01844-2

[ece38453-bib-0064] Lotka, A. (1925). Elements of physical biology. Williams and Wilkins.

[ece38453-bib-0065] Martignoni, M. M. , Garnier, J. , Zhang, X. , Rosa, D. , Kokkoris, V. , Tyson, R. C. , & Hart, M. M. (2021). Co‐inoculation with arbuscular mycorrhizal fungi differing in carbon sink strength induces a synergistic effect in plant growth. Journal of Theoretical Biology, 531, 110859. 10.1016/j.jtbi.2021.110859 34389360

[ece38453-bib-0066] Martignoni, M. M. , Hart, M. M. , Garnier, J. , & Tyson, R. C. (2020). Parasitism within mutualist guilds explains the maintenance of diversity in multi‐species mutualisms. Theoretical Ecology, 13, 615–627. 10.1007/s12080-020-00472-9

[ece38453-bib-0067] May, R. M. (1972). Will a large complex system be stable? Nature, 238, 413. 10.1038/238413a0 4559589

[ece38453-bib-0068] May, R. M. (1973). Qualitative stability in model ecosystems. Ecology, 54, 638–641. 10.2307/1935352

[ece38453-bib-0069] May, R. M. (1976). Models of two interacting populations. In R. M. May (Ed.), Theoretical ecology. Blackwell Scientific Publications.

[ece38453-bib-0070] May, R. M. (1978). Mathematical aspects of the dynamics of animal populations. In S. A. Levin (Ed.), Studies in mathematical biology (pp. 342–343). Blackwell Scientific.

[ece38453-bib-0071] McCann, K. S. , & Gellner, G. (2020). Theoretical ecology: concepts and applications. Oxford University Press.

[ece38453-bib-0072] McGill, B. (2005). A mechanistic model of a mutualism and its ecological and evolutionary dynamics. Ecological Modelling, 187, 413–425. 10.1016/j.ecolmodel.2005.02.002

[ece38453-bib-0073] Meyer, J. S. , Tsuchiya, H. M. , & Fredrickson, A. G. (1975). Dynamics of mixed populations having complementary metabolism. Biotechnology and Bioengineering, 17, 1065–1081. 10.1002/bit.260170709

[ece38453-bib-0074] Mittelbach, G. G. , & McGill, B. J. (2019). Community ecology. Oxford University Press.

[ece38453-bib-0075] Mohammed, M. M. A. , Landi, P. , Minoarivelo, H. O. , & Hui, C. (2018). Frugivory and seed dispersal: Extended bi‐stable persistence and reduced clustering of plants. Ecological Modelling, 380, 31–39. 10.1016/j.ecolmodel.2018.04.010

[ece38453-bib-0076] Moore, C. M. , Catella, S. A. , & Abbott, K. C. (2018). Population dynamics of mutualism and intraspecific density dependence: How θ‐logistic density dependence affects mutualistic positive feedback. Ecological Modelling, 368, 191–197. 10.1016/j.ecolmodel.2017.11.016

[ece38453-bib-0077] Morales, M. A. , Morris, W. F. , & Wilson, W. G. (2008). Allee dynamics generated by protection mutualisms can drive oscillations in trophic cascades. Theoretical Ecology, 1, 77–88. 10.1007/s12080-007-0006-9

[ece38453-bib-0078] Morin, P. J. (2011). Mutualisms. In Community ecology (pp. 166–186). Blackwell Publishing Ltd.

[ece38453-bib-0079] Mougi, A. , & Kondoh, M. (2012). Diversity of interaction types and ecological community stability. Science, 337, 349–351. 10.1126/science.1220529 22822151

[ece38453-bib-0080] Neuhauser, C. , & Fargione, J. E. (2004). A mutualism‐parasitism continuum model and its application to plant‐mycorrhizae interactions. Ecological Modelling, 177, 337–352. 10.1016/j.ecolmodel.2004.02.010

[ece38453-bib-0081] Okuyama, T. , & Holland, J. N. (2008). Network structural properties mediate the stability of mutualistic communities. Ecology Letters, 11, 208–216. 10.1111/j.1461-0248.2007.01137.x 18070101

[ece38453-bib-0082] Palmer, T. M. , Doak, D. F. , Stanton, M. L. , Bronstein, J. L. , Kiers, E. T. , Young, T. P. , Goheen, J. R. , & Pringle, R. M. (2010). Synergy of multiple partners, including freeloaders, increases host fitness in a multispecies mutualism. Proceedings of the National Academy of Sciences of the United States of America, 107, 17234–17239. 10.1073/pnas.1006872107 20855614 PMC2951420

[ece38453-bib-0083] Parker, M. A. (2001). Mutualism as a constraint on invasion success for legumes and rhizobia. Diversity and Distributions, 7, 125–136. 10.1046/j.1472-4642.2001.00103.x

[ece38453-bib-0084] Pierce, N. E. , & Young, W. R. (1986). Lycaenid butterflies and ants: two‐species stable equilibria in mutualistic, commensal, and parasitic interactions. American Naturalist, 128, 216–227. 10.1086/284555

[ece38453-bib-0085] Raerinne, J. (2020). Ghosts of competition and predation past: Why ecologists value negative over positive interactions. The Bulletin of the Ecological Society of America, 101(4), e01766. 10.1002/bes2.1766

[ece38453-bib-0086] Rai, B. , Freedman, H. I. , & Addicott, J. F. (1983). Analysis of three species models of mutualism in predator‐prey and competitive systems. Mathematical Biosciences, 65, 13–50. 10.1016/0025-5564(83)90069-X

[ece38453-bib-0087] Revilla, T. A. (2015). Numerical responses in resource‐based mutualisms: A time scale approach. Journal of Theoretical Biology, 378, 39–46. 10.1016/j.jtbi.2015.04.012 25936757

[ece38453-bib-0088] Ringel, M. S. , Hu, H. H. , Anderson, G. , & Ringel, M. S. (1996). The stability and persistence of mutualisms embedded in community interactions. Theoretical Population Biology, 50, 281–297. 10.1006/tpbi.1996.0032 9000491

[ece38453-bib-0089] Rosenzweig, M. L. (1971). Paradox of enrichment: Destabilization of exploitation ecosystems in ecological time. Science, 171, 385–387. 10.1126/science.171.3969.385 5538935

[ece38453-bib-0090] Scheuring, I. (1992). “The orgy of mutualism” as an artefact: a stage structured model of plant‐pollinator and seed‐dispersal systems. Abstracta Botanica, 16, 65–70.

[ece38453-bib-0091] Schupp, E. W. , Jordano, P. , & Gómez, J. M. (2017). A general framework for effectiveness concepts in mutualisms. Ecology Letters, 20, 577–590. 10.1111/ele.12764 28349589

[ece38453-bib-0092] Soberón, J. M. , & Martinez del Rio, C. (1981). The dynamics of a plant‐pollinator interaction. Journal of Theoretical Biology, 91, 363–378. 10.1016/0022-5193(81)90238-1

[ece38453-bib-0093] Thébault, E. , & Fontaine, C. (2010). Stability of ecological communities and the architecture of mutualistic and trophic networks. Science, 329, 853–856. 10.1126/science.1188321 20705861

[ece38453-bib-0094] Thompson, A. R. , Nisbet, R. M. , & Schmitt, R. J. (2006). Dynamics of mutualist populations that are demographically open. Journal of Animal Ecology, 75, 1239–1251. 10.1111/j.1365-2656.2006.01145.x 17032356

[ece38453-bib-0095] Thompson, J. N. (1988). Variation in interspecific interactions. Annual Review of Ecology and Systematics, 19, 65–87. 10.1146/annurev.es.19.110188.000433

[ece38453-bib-0096] Tonkyn, D. W. (1986). Predator‐mediated mutualism: Theory and tests in the Homoptera. Journal of Theoretical Biology, 118, 15–31. 10.1016/S0022-5193(86)80005-4

[ece38453-bib-0097] Travis, C. C. , & Post, W. M. (1979). Dynamics and comparative statics of mutualistic communities. Journal of Theoretical Biology, 78, 553–571. 10.1016/0022-5193(79)90190-5 513796

[ece38453-bib-0098] Turchin, P. (2003). Complex population dynamics. Princeton University Press.

[ece38453-bib-0099] Valdovinos, F. S. (2019). Mutualistic networks: Moving closer to a predictive theory. Ecology Letters, 22, 1517–1534. 10.1111/ele.13279 31243858

[ece38453-bib-0100] Valdovinos, F. S. , Berlow, E. L. , Moisset De Espanés, P. , Ramos‐Jiliberto, R. , Vázquez, D. P. , & Martinez, N. D. (2018). Species traits and network structure predict the success and impacts of pollinator invasions. Nature Communications, 9, 1–8. 10.1038/s41467-018-04593-y PMC5981428 29855466

[ece38453-bib-0101] Valdovinos, F. S. , Brosi, B. J. , Briggs, H. M. , Moisset de Espanés, P. , Ramos‐Jiliberto, R. , & Martinez, N. D. (2016). Niche partitioning due to adaptive foraging reverses effects of nestedness and connectance on pollination network stability. Ecology Letters, 19, 1277–1286. 10.1111/ele.12664 27600659

[ece38453-bib-0102] Valdovinos, F. S. , & Marsland, R. (2021). Niche theory for mutualism: A graphical approach to plant‐pollinator network dynamics. American Naturalist, 197, 393–404. 10.1086/712831 33755542

[ece38453-bib-0103] Valdovinos, F. S. , Moisset de Espanés, P. , Flores, J. D. , & Ramos‐Jiliberto, R. (2013). Adaptive foraging allows the maintenance of biodiversity of pollination networks. Oikos, 122, 907–917. 10.1111/j.1600-0706.2012.20830.x

[ece38453-bib-0104] van der Heijden, M. G. A. , Bardgett, R. D. , & van Straalen, N. M. (2008). The unseen majority: Soil microbes as drivers of plant diversity and productivity in terrestrial ecosystems. Ecology Letters, 11, 296–310. 10.1111/j.1461-0248.2007.01139.x 18047587

[ece38453-bib-0105] Vandermeer, J. H. , & Boucher, D. H. (1978). Varieties of mutualistic interaction in population models. Journal of Theoretical Biology, 74, 549–558. 10.1016/0022-5193(78)90241-2 732344

[ece38453-bib-0106] Vandermeer, J. H. , & Goldberg, D. E. (2013). Population ecology: First principles. Princeton University Press.

[ece38453-bib-0107] Vázquez, D. P. , Ramos‐Jiliberto, R. , Urbani, P. , & Valdovinos, F. S. (2015). A conceptual framework for studying the strength of plant‐animal mutualistic interactions. Ecology Letters, 18, 385–400. 10.1111/ele.12411 25735791

[ece38453-bib-0108] Volterra, V. (1926). Variazioni e fluttuazioni del numero d’individui in specie animali convivienti. Memoroo Acadamei Linceii, 2, 31–113.

[ece38453-bib-0109] Wang, Y. (2019). Dynamics of a plant‐nectar‐pollinator model and its approximate equations. Mathematical Biosciences, 307, 42–52. 10.1016/j.mbs.2018.12.001 30528332

[ece38453-bib-0110] Wells, H. (1983). Population equilibria and stability in plant‐animal pollination systems. Journal of Theoretical Biology, 100, 685–699. 10.1016/0022-5193(83)90330-2

[ece38453-bib-0111] Whittaker, R. H. (1975). Symbiosis. In Communities and ecosystems (pp. 37–42). MacMillan.

[ece38453-bib-0112] Williamson, M. H. (1972). The analysis of biological populations (pp. 94–99). Edward Arnold.

[ece38453-bib-0113] Wolin, C. L. (1985). The population dynamics of mutualistic systems. In D. H. Boucher (Ed.), The biology of mutualism (pp. 248–269). Oxford University Press.

[ece38453-bib-0114] Wolin, C. L. , & Lawlor, L. R. (1984). Models of facultative mutualism: Density effects. The American Naturalist, 124, 843–862. 10.1086/284320

[ece38453-bib-0115] Wotton, D. M. , & Kelly, D. (2011). Frugivore loss limits recruitment of large‐seeded trees. Proceedings of the Royal Society B: Biological Sciences, 278, 3345–3354. 10.1098/rspb.2011.0185 PMC3177627 21450732

[ece38453-bib-0116] Wright, D. H. (1989). A simple, stable model of mutualism incorporating handling time. American Naturalist, 134, 664–667. 10.1086/285003

[ece38453-bib-0117] Wu, F. , Lopatkin, A. J. , Needs, D. A. , Lee, C. T. , Mukherjee, S. , & You, L. (2019). A unifying framework for interpreting and predicting mutualistic systems. Nature Communications, 10, 1–10. 10.1038/s41467-018-08188-5 PMC6335432 30651549

[ece38453-bib-0118] Zhang, Z. (2003). Mutualism or cooperation among competitors promotes coexistence and competitive ability. Ecological Modelling, 164, 271–282. 10.1016/S0304-3800(03)00069-3

