# Correction to Ecological theory of mutualism: Robust patterns of stability and thresholds in two‐species population models

**DOI:** 10.1002/ece3.70007

**Published:** 2024-07-18

Hale, K. R. S. & Valdovinos, F. S. (2021). Ecological theory of mutualism: Robust patterns of stability and thresholds in two‐species population models. Ecology and Evolution 11:17651–17671. doi:10.1002/ece3.8453

In the caption of Figure 1, the interaction strengths listed for panels (b) and (d) were switched. The text “When both mutualists are obligate upon their partner (*N*_*i*_ = *K*_*i*_ ≤ 0 when *N*_*j*_ = 0) and benefits are weak, the system exhibits a threshold in density above which species exhibit unbounded growth and below which extinctions occur (b), whereas if benefits are strong, only extinctions occur (d).” was incorrect. The text should read “When both mutualists are obligate upon their partner (*N*_*i*_ = *K*_*i*_ ≤ 0 when *N*_*j*_ = 0) and benefits are strong, the system exhibits a threshold in density above which species exhibit unbounded growth and below which extinctions occur (b), whereas if benefits are weak, only extinctions occur (d).”

We apologize for this error.

