# Supplementary material for: Ecological theory of mutualism: Robust patterns of stability and thresholds in two‐species population models
Source: Ecol Evol. 2021 Dec 15;11(24):17651–71. doi: 10.1002/ece3.8453 (PMC8717353; doi:10.1002/ece3.8453)
Supplement: Supplementary file 1 — Table S1 [file ECE3-11-17651-s001.docx]

# Supplementary Information

**Table S1. Models of pairwise mutualism.** Equations largely follow the notation from the original citations. All parameters are positive (> 0) unless otherwise specified. Models with unique mathematical forms are given unique equation numbers, starting chronologically (compared to the selected models from main text). We encourage the readers to refer to the original references for the model derivations and interpretation of parameters. Notes include inspiring system and obligacy, if specified by authors. We use $\boldsymbol{N}_{i}^{'}$ to notate discrete-time models, and $d\boldsymbol{N}_{i}/dt$to notate continuous-time models.

| **Reference** | **Eqn** | **Models for Pairwise Mutualism** ($i = 1, 2$) | **Notes** |
| --- | --- | --- | --- |
| Gause & Witt 1935 | S1 | $\frac{d\boldsymbol{N}_{i}}{dt}=r_{i}\boldsymbol{N}_{i}\left( \frac{K_{i}+\alpha_{ij}\boldsymbol{N}_{j}-\boldsymbol{N}_{i}}{K_{i}} \right)$ | Facultative only |
| Whittaker 1975 | S2  S1 | $\left\{ \begin{aligned} \frac{d\boldsymbol{N}_{1}}{dt}=r_{1}\boldsymbol{N}_{1}\left( \frac{K_{1}+\alpha_{12}\boldsymbol{N}_{2}-\boldsymbol{N}_{1}}{K_{1}+\alpha_{12}\boldsymbol{N}_{2}} \right) \\ \frac{d\boldsymbol{N}_{2}}{dt}={r_{2}\boldsymbol{N}}_{2}\left( \frac{K_{2}+\alpha_{21}\boldsymbol{N}_{1}-\boldsymbol{N}_{2}}{K_{2}} \right) \end{aligned} \right.$ | Symbiont ($\boldsymbol{N}_{1}$)-Host ($\boldsymbol{N}_{2}$)  Obligate$\boldsymbol{N}_{1}$ when $K_{1}=0$ Parasitism when $\alpha_{21}<0$ |
|  | S2  S3 | $\left\{ \begin{aligned} \frac{d\boldsymbol{N}_{1}}{dt}=r_{1}\boldsymbol{N}_{1}\left( \frac{\alpha_{12}\boldsymbol{N}_{2}-\boldsymbol{N}_{1}}{\alpha_{12}\boldsymbol{N}_{2}} \right) \\ \frac{d\boldsymbol{N}_{2}}{dt}=\frac{{r_{2}\boldsymbol{N}}_{2}}{K_{2}}\left( K_{2}+\frac{aD\boldsymbol{N}_{1}}{D+\boldsymbol{N}_{2}}-\boldsymbol{N}_{2} \right) \end{aligned} \right.$ | Obligate Symbiont ($\boldsymbol{N}_{1}$)-Host ($\boldsymbol{N}_{2}$)  Arbitrary external factors limit benefit to $\boldsymbol{N}_{2}$ |
| May 1976 | S4  S2 | $\left\{ \begin{aligned} \frac{d\boldsymbol{N}_{1}}{dt}=\boldsymbol{N}_{1}\left( \frac{I\boldsymbol{N}_{2}}{CD+C\boldsymbol{N}_{1}+\boldsymbol{N}_{1}\boldsymbol{N}_{2}}-d_{1} \right) \\ \frac{d\boldsymbol{N}_{2}}{dt}=r_{2}\boldsymbol{N}_{2}\left( \frac{\alpha_{21}\boldsymbol{N}_{1}-\boldsymbol{N}_{2}}{\alpha_{21}\boldsymbol{N}_{1}} \right) \end{aligned} \right.$ | Plant ($\boldsymbol{N}_{1}$)-Pollinator ($\boldsymbol{N}_{2}$) and others  Obligate only |
| Vandermeer & Boucher 1978 | S1 | $\frac{d\boldsymbol{N}_{i}}{dt}=\boldsymbol{N}_{i}(r_{i}+\alpha_{ij}\boldsymbol{N}_{j}-\alpha_{ii}\boldsymbol{N}_{i})$ | Legume ($\boldsymbol{N}_{1}$)-*Rhizobium* ($\boldsymbol{N}_{2}$)  Obligate when $K_{i}=r_{i}/\alpha_{ii}\leq0$ |
| Addicott 1981 | S5 | $\frac{d\boldsymbol{N}_{i}}{dt}=r_{i}\left( \frac{K_{i}-\boldsymbol{N}_{i}}{K_{i}} \right)\left( 1+\frac{\alpha_{ij}\boldsymbol{N}_{j}}{K_{i}} \right)$ | Aphid ($\boldsymbol{N}_{1}$)-Ant ($\boldsymbol{N}_{2}$)  Facultative only |
|  | S6 | $\frac{d\boldsymbol{N}_{i}}{dt}={r_{i}\boldsymbol{N}}_{i}\left( \frac{K_{i}-\boldsymbol{N}_{i}}{K_{i}} \right)\left( 1+\frac{\alpha_{ij}\boldsymbol{N}_{j}}{K_{i}} \right)$ | *Reference reports Eqn S5. Figures suggest Eqn S6 & subsequent authors use Eqn S6 |
| Soberón & Martinez del Rio 1981 | S7  S8 | $\left\{ \begin{aligned} \frac{d\boldsymbol{N}_{1}}{dt}=\boldsymbol{N}_{1}\left( \frac{k_{1}\sigma\mu\boldsymbol{N}_{2}}{1+\sigma\phi\mu^{2}\boldsymbol{N}_{1}}-\gamma\right) \\ \frac{d\boldsymbol{N}_{2}}{dt}=\boldsymbol{N}_{2}\left( \epsilon\left( K_{2}-\boldsymbol{N}_{2} \right)+\frac{k_{2}\sigma\mu^{2}\boldsymbol{N}_{1}}{1+\sigma\phi\mu^{2}\boldsymbol{N}_{1}} \right) \end{aligned} \right.$ | Plant ($\boldsymbol{N}_{1}$)-Pollinator ($\boldsymbol{N}_{2}$)  Obligate $\boldsymbol{N}_{1}$; obligate $\boldsymbol{N}_{2}$ when $K_{2}=0$ |
| Gilpin *et al.* 1982 |  | $\boldsymbol{N}_{i}^{'}=\boldsymbol{N}_{i}\left( 1+r_{i}-{r_{i}\boldsymbol{N}}_{i}-r_{i}a_{ij}\boldsymbol{N}_{j} \right)$ | Mutualism when $a_{ij}<0$, competition when $a_{ij}>0$ |
| Dean 1983 | S9 | $\frac{d\boldsymbol{N}_{i}}{dt}=r_{i}\boldsymbol{N}_{i}\left( \frac{{k_{i}\boldsymbol{-N}}_{i}}{k_{i}} \right)$  $k_{i}=K_{i_{max}}\left( 1-e^{-(a_{i}\boldsymbol{N}_{j}+C_{i})/K_{i_{max}}} \right)$ | Unintended behaviors; updated by Graves *et al.* 2006 |
| Wells 1983 | S10  S11 | $\left\{ \begin{aligned} \frac{d\boldsymbol{N}_{1}}{dt}=\boldsymbol{N}_{1}\left( \frac{b\boldsymbol{N}_{2}}{a\boldsymbol{N}_{1}+\boldsymbol{N}_{2}+c}-df\boldsymbol{N}_{1}-d \right) \\ \frac{d\boldsymbol{N}_{2}}{dt}=\boldsymbol{N}_{2}\left( \frac{m\boldsymbol{N}_{1}}{r\boldsymbol{N}_{2}+\boldsymbol{N}_{1}+h}-g \right) \end{aligned} \right.$ | Plant ($\boldsymbol{N}_{1}$)-Pollinator ($\boldsymbol{N}_{2})$  Obligate only |
| Wolin & Lawlor 1984 | S12 | $\frac{d\boldsymbol{N}_{i}}{dt}=\boldsymbol{N}_{i}\left( r_{i}-\frac{b\boldsymbol{N}_{i}}{1+m\boldsymbol{N}_{j}}-d\boldsymbol{N}_{i} \right)$ | Multiple models (including S2, S6) where benefits are a fn. of intra-specific limitation in birth ($b$) & death ($d$) processes Facultative only  Reduces $b$ to at most 0 |
|  | S13 | $\frac{d\boldsymbol{N}_{i}}{dt}=\boldsymbol{N}_{i}\left( r_{i}-(b-m\boldsymbol{N}_{j}+d)\boldsymbol{N}_{i} \right)$ | Reduces $b$ without limit |
|  | S14 | $\frac{d\boldsymbol{N}_{i}}{dt}=\boldsymbol{N}_{i}\left( r_{i}\left( 1-\frac{\boldsymbol{N}_{i}}{K} \right)+m\boldsymbol{N}_{j}e^{-\alpha\boldsymbol{N}_{i}} \right)$ | Benefits decrease exponentially |
| Tonkyn 1986 | S15 | $\frac{d\boldsymbol{N}_{i}}{dt}=r_{i}\boldsymbol{N}_{i}\left( 1-\left( \frac{\boldsymbol{N}_{i}+\alpha_{ij}\boldsymbol{N}_{j}-U_{i}}{K_{i}-U_{i}} \right)^{2} \right)$ | Feeding aggregations among phloem-feeding insects  Competition at high $\boldsymbol{N}_{i}$ or $\boldsymbol{N}_{j}$ |
| Pierce & Young 1986 | S16  S17 | $\left\{ \begin{aligned} \frac{d\boldsymbol{N}_{1}}{dt}=\boldsymbol{N}_{1}\left( r_{1}(\boldsymbol{N}_{2})-\frac{\boldsymbol{N}_{1}}{K_{1}} \right) \\ \frac{d\boldsymbol{N}_{2}}{dt}=r_{2}\boldsymbol{N}_{2}\left( 1-\frac{\boldsymbol{N}_{2}}{K_{2}(\boldsymbol{N}_{1})} \right) \end{aligned} \right.$ | Lycaenid ($\boldsymbol{N}_{1}$)-Ant ($\boldsymbol{N}_{2}$)  $r_{1}\left( \boldsymbol{N}_{2} \right)$ is 1’s growth rate as a fn. of $\boldsymbol{N}_{2}$; $K_{2}(\boldsymbol{N}_{1})$ is 2’s equilibrium density of 2 as a fn. of $\boldsymbol{N}_{1}$. Parasitism when $K_{2}(\boldsymbol{N}_{1})$ is decreasing |
| Wright 1989 | S18 | $\frac{d\boldsymbol{N}_{i}}{dt}=\boldsymbol{N}_{i}\left( r_{i}(1-c_{i}\boldsymbol{N}_{i})+b_{ij}\frac{a_{ij}\boldsymbol{N}_{j}}{1+a_{ij}h_{ij}\boldsymbol{N}_{j}} \right)$ | Pollinators & other forager mutualists |
|  | S8 | $\frac{d\boldsymbol{N}_{i}}{dt}=\boldsymbol{N}_{i}\left( r_{i}-c_{i}\boldsymbol{N}_{i}+b_{ij}\frac{a_{ij}\boldsymbol{N}_{j}}{1+a_{ij}h_{ij}\boldsymbol{N}_{j}} \right)$ | *Reference reports Eqn S18. Figures suggest Eqn S8 & subsequent authors use Eqn S8  Obligate when $r_{i}<0$ |
| Hernandez 1998 | S19 | $\frac{d\boldsymbol{N}_{i}}{dt}=r_{i}\boldsymbol{N}_{i}\left( 1-\frac{\boldsymbol{N}_{i}}{K_{i}}+\left( \frac{b_{i}\boldsymbol{N}_{j}-c_{i}\boldsymbol{N}_{j}^{2}}{1+d_{i}\boldsymbol{N}_{j}^{2}} \right)\frac{\boldsymbol{N}_{j}}{K_{i}} \right)$ | Facultative  Parasitism at high $\boldsymbol{N}_{j}$ |
|  | S20 | $\frac{d\boldsymbol{N}_{i}}{dt}=r_{i}\boldsymbol{N}_{i}\left( -1+\left( \frac{b_{i}\boldsymbol{N}_{j}-c_{i}\boldsymbol{N}_{j}^{2}}{1+d_{i}\boldsymbol{N}_{j}^{2}} \right)\frac{\boldsymbol{N}_{j}}{K_{i}} \right)$ | Obligate  Parasitism at high $\boldsymbol{N}_{j}$ |
| Parker 2001 |  | $\left\{ \begin{aligned} \boldsymbol{N}_{1}^{'}=\boldsymbol{N}_{1}\left( 1+r-rc\boldsymbol{N}_{1}+\frac{m_{1}\theta}{1+e^{-u(\boldsymbol{N}_{2}-B_{o})}} \right) \\ \boldsymbol{N}_{2}^{'}=\boldsymbol{N}_{2}\left( 1+s-sd\boldsymbol{N}_{2} \right)+\boldsymbol{N}_{1}\left( \frac{m_{2}\theta}{1+e^{-u\left( \boldsymbol{N}_{2}-B_{o} \right)}} \right) \end{aligned} \right.$ | Legume ($\boldsymbol{N}_{1}$)-*Rhizobium* ($\boldsymbol{N}_{2}$)  Facultative only |
| Holland *et al.* 2002 | S21  S22 | $\left\{ \begin{aligned} \frac{d\boldsymbol{N}_{1}}{dt}=\boldsymbol{N}_{1}\left( \left( 1-a \right)\alpha F\left( 1-e^{\left( -\frac{\gamma_{1}\boldsymbol{N}_{2}}{F\boldsymbol{N}_{1}} \right)} \right)\left( e^{\left( -\frac{\gamma_{2}\boldsymbol{N}_{2}}{F\boldsymbol{N}_{1}} \right)} \right)-d_{1}-g\boldsymbol{N}_{1} \right) \\ \frac{d\boldsymbol{N}_{2}}{dt}=\left( 1-a \right)\alpha F\left( 1-e^{\left( -\frac{\gamma_{1}\boldsymbol{N}_{2}}{F\boldsymbol{N}_{1}} \right)} \right)\left( e^{\left( -\frac{\gamma_{2}\boldsymbol{N}_{2}}{F\boldsymbol{N}_{1}} \right)} \right)-d_{2}\boldsymbol{N}_{2} \end{aligned} \right.$ | Obligate Senita Cactus ($\boldsymbol{N}_{1}$) – Moth ($\boldsymbol{N}_{2}$)  Detailed application of more general theory |
| Zhang 2003 | S23 | $\frac{d\boldsymbol{N}_{i}}{dt}=R_{i}\boldsymbol{N}_{i}\left( c_{i}-\boldsymbol{N}_{i}-a_{i}\left( \boldsymbol{N}_{j}-b_{i} \right)^{2} \right)$ | Interactions between species at the same trophic level  Competition at high $\boldsymbol{N}_{j}$  $-{\infty<b}_{i}<\infty$ |
| Neuhauser & Fargione 2004 | S24  S1 | $\left\{ \begin{aligned} \frac{d\boldsymbol{N}_{1}}{dt}=r_{1}\boldsymbol{N}_{1}\left( 1-\frac{\boldsymbol{N}_{1}}{K_{1}+\gamma_{12}\boldsymbol{N}_{2}}-a\boldsymbol{N}_{2} \right) \\ \frac{d\boldsymbol{N}_{2}}{dt}={r_{2}\boldsymbol{N}}_{2}\left( \frac{K_{2}+\alpha_{21}\boldsymbol{N}_{1}-\boldsymbol{N}_{2}}{K_{2}} \right) \end{aligned} \right.$ | Plant ($\boldsymbol{N}_{1}$)-Mycorrhizae ($\boldsymbol{N}_{2}$)  Facultative only |
| McGill 2005 | S25 | $\boldsymbol{N}_{i}^{'}=\boldsymbol{N}_{i}\left( 1+W_{i} \right)$  where ${W_{i}=r}_{i}*\left( 2\left[ 2\left( 1+e^{-c\left( R_{ii}-u_{ii}\boldsymbol{N}_{i}-p_{ii}u_{ii}\boldsymbol{N}_{i} \right)} \right)*\left( 1+e^{-c\left( R_{ji}-u_{ji}\boldsymbol{N}_{i}-{\epsilon p}_{ji}u_{jj}\boldsymbol{N}_{j} \right)} \right) \right]^{-1}-1 \right)$ | Plant ($\boldsymbol{N}_{1}$)-Mycorrhizae ($\boldsymbol{N}_{2}$)  Facultative only  $R_{ij}$ is resource type $i$ available to species $j$per unit time |
| Graves *et al.* 2006 | S9 | $\frac{d\boldsymbol{N}_{i}}{dt}=\boldsymbol{N}_{i}\left( r_{i0}+(r_{i1}-r_{i0})\left( 1-e^{-k_{i}\boldsymbol{N}_{j}} \right)-a_{i}\boldsymbol{N}_{i} \right)$ | Lichens and other N-fixing symbioses  Updates Dean’s (1983) model so that mutualism affects per-capita growth rate instead of equilb. density directly. Obligate when $r_{i0}<0,r_{i1}+r_{i0}>0$ |
| Thompson *et al.* 2006 | S26 | $\frac{d\boldsymbol{N}_{i}}{dt}=\left( \rho_{i}\left( b_{i}\boldsymbol{N}_{i}+\frac{\alpha_{i}\boldsymbol{N}_{j}}{\beta_{i}+\boldsymbol{N}_{j}}\boldsymbol{N}_{i} \right)+I_{i} \right)\left( 1-\frac{\boldsymbol{N}_{i}}{S_{i}} \right)-d_{i_{max}}\boldsymbol{N}_{i}$ | Set of models with different combinations of benefit. Closed system when $I_{i}=0$, $\rho_{i}=1$. Obligate when $\rho_{i}b_{i}<d_{i_{max}}$  Benefit: increases birth |
|  | S27 | $\frac{d\boldsymbol{N}_{i}}{dt}=\left( \rho_{i}b_{i}\boldsymbol{N}_{i}+I_{i} \right)\left( 1-\frac{\boldsymbol{N}_{i}}{S_{i}} \right)-\left( d_{i_{min}}+\frac{{d_{i}}_{diff}}{1+c_{i}\boldsymbol{N}_{j}} \right)\boldsymbol{N}_{i}$ | Decreases death |
|  | S28 | $\frac{d\boldsymbol{N}_{i}}{dt}=\left( \rho_{i}b_{i}\boldsymbol{N}_{i}+I_{i} \right)\left( 1-\frac{\boldsymbol{N}_{i}}{S_{i}+\boldsymbol{N}_{j}} \right)-d_{i_{max}}\boldsymbol{N}_{i}$ | Increases carrying capacity |
| Holland & DeAngelis 2010 | S29 | $\frac{d\boldsymbol{N}_{i}}{dt}=\boldsymbol{N}_{i}\left( r_{i}+c_{i}\left( \frac{a_{ij}\boldsymbol{N}_{j}}{h_{j}+\boldsymbol{N}_{j}} \right)-q_{i}\left( \frac{\beta_{ij}\boldsymbol{N}_{j}}{e_{i}+\boldsymbol{N}_{i}} \right)-s_{i}\boldsymbol{N}_{i} \right)$ | Bidirectional Consumer -Resource, e.g., Plant ($\boldsymbol{N}_{1}$)-Mycorrhizae ($\boldsymbol{N}_{2}$) |
|  | S29  S8 | $\left\{ \begin{aligned} \frac{d\boldsymbol{N}_{1}}{dt}=\boldsymbol{N}_{1}\left( r_{1}+c_{1}\left( \frac{a_{12}\boldsymbol{N}_{2}}{h_{2}+\boldsymbol{N}_{2}} \right)-q_{1}\left( \frac{\beta_{12}\boldsymbol{N}_{2}}{e_{1}+\boldsymbol{N}_{1}} \right)-s_{1}\boldsymbol{N}_{1} \right) \\ \frac{d\boldsymbol{N}_{2}}{dt}=\boldsymbol{N}_{2}\left( r_{2}+c_{2}\left( \frac{a_{21}\boldsymbol{N}_{1}}{h_{1}+\boldsymbol{N}_{1}} \right)-s_{2}\boldsymbol{N}_{2} \right) \end{aligned} \right.$ | Unidirectional, e.g., Plant ($\boldsymbol{N}_{1}$)-Pollinator ($\boldsymbol{N}_{2}$)  Obligate when $r_{i}=0$ |
| Fishman & Hadany 2010 | S10  S11 | $\left\{ \begin{aligned} \frac{d\boldsymbol{N}_{1}}{dt}=\boldsymbol{N}_{1}\left( \frac{\eta\alpha\boldsymbol{N}_{2}}{1+\alpha\boldsymbol{N}_{1}+\alpha\beta\boldsymbol{N}_{2}}-b-c\boldsymbol{N}_{1} \right) \\ \frac{d\boldsymbol{N}_{2}}{dt}=\boldsymbol{N}_{2}\left( \frac{\mu\alpha\boldsymbol{N}_{1}}{1+\alpha\boldsymbol{N}_{1}+\alpha\beta\boldsymbol{N}_{2}}-d \right) \end{aligned} \right.$ | Plant ($\boldsymbol{N}_{1}$)-Pollinator ($\boldsymbol{N}_{2}$)  Obligate only  Approx. from individual-based model |
| Kang *et al.* 2011 | S30  S1 | $\left\{ \begin{aligned} \frac{d\boldsymbol{N}_{1}}{dt}=\boldsymbol{N}_{1}\left( r_{f}\left( \frac{a\boldsymbol{N}_{2}^{2}}{b+a\boldsymbol{N}_{2}^{2}} \right)-r_{c}\boldsymbol{N}_{2}-d_{1}\boldsymbol{N}_{1} \right) \\ \frac{d\boldsymbol{N}_{2}}{dt}=\boldsymbol{N}_{2}(r_{a}\boldsymbol{N}_{1}-d_{2}\boldsymbol{N}_{2}) \end{aligned} \right.$ | Fungal garden ($\boldsymbol{N}_{1}$)-Leaf cutter ant ($\boldsymbol{N}_{2}$)  Obligate only |
| Johnson & Amarasekare 2013 | S31 | $\frac{d\boldsymbol{N}_{i}}{dt}=\boldsymbol{N}_{i}\left( r_{i}+\frac{m_{ij}\boldsymbol{N}_{j}}{{1+m}_{ij}\tau_{i}\boldsymbol{N}_{j}+\alpha_{i}\boldsymbol{N}_{i}^{2}} \right)$ | Obligate when $r_{i}<0$ |
| García-Algarra *et al.* 2014 | S32 | $\frac{d\boldsymbol{N}_{i}}{dt}=\boldsymbol{N}_{i}(r_{i}+b_{ij}\boldsymbol{N}_{j}-(\alpha_{i}+c_{i}b_{ij}\boldsymbol{N}_{j})\boldsymbol{N}_{i})$ | Obligate when $r_{i}\leq0$ |
| Revilla 2015 | S33 | $\frac{d\boldsymbol{N}_{i}}{dt}=\boldsymbol{N}_{i}\left( r_{i}+\frac{\sigma_{i}\beta_{i}\alpha_{j}\boldsymbol{N}_{j}}{\omega_{j}+\beta_{i}\boldsymbol{N}_{i}}-c_{i}\boldsymbol{N}_{i} \right)$ | Assuming steady-state reward dynamics  Exchanges of resources for resources, e.g., Lichens  Obligate when $r_{i}\leq0$ |
|  | S34  S33 | $\left\{ \begin{aligned} \frac{d\boldsymbol{N}_{1}}{dt}=\boldsymbol{N}_{1}\left( r_{1}+\frac{\sigma_{0}\beta_{0}\alpha_{0}N_{0}+\sigma_{1}\beta\alpha\boldsymbol{N}_{2}}{\omega+\beta_{0}N_{0}+\beta\boldsymbol{N}_{2}}-c_{1}\boldsymbol{N}_{1} \right) \\ \frac{d\boldsymbol{N}_{2}}{dt}=\boldsymbol{N}_{2}\left( r_{2}+\frac{\sigma_{2}\beta\alpha\boldsymbol{N}_{1}}{\omega+\beta_{0}N_{0}+\beta\boldsymbol{N}_{2}}-c_{2}\boldsymbol{N}_{2} \right) \end{aligned} \right.$ | Exchanges of resources for services, e.g., Plant ($\boldsymbol{N}_{1}$)-Disperser ($\boldsymbol{N}_{2}$)  Obligate when $r_{i}\leq0$ |
| Moore *et al.* 2018 | S35 | $\frac{d\boldsymbol{N}_{i}}{dt}=\boldsymbol{N}_{i}(r_{i}+\beta_{j}\boldsymbol{N}_{j}-\alpha_{i}\boldsymbol{N}_{i}^{\theta_{i}})$ | Accelerating intraspecific density-dependence when $\theta_{i}>1$; Eqn S1 when $\theta_{i}=1$  Facultative only ($r_{i}>0$) |
| Cropp & Norbury 2019 | S36 | $\frac{d\boldsymbol{N}_{i}}{dt}=\boldsymbol{N}_{i}\left( r_{i}\left( \frac{\boldsymbol{N}_{j}}{\varepsilon_{j}+\boldsymbol{N}_{j}} \right)\boldsymbol{R}+a_{ij}\boldsymbol{N}_{j}-a_{ii}\boldsymbol{N}_{i} \right)$  where $\boldsymbol{R=}1-\boldsymbol{N}_{1}-\boldsymbol{N}_{2}$ | Autotrophs ($\boldsymbol{N}_{1}$)-($\boldsymbol{N}_{2}$)  Conservation of mass limits a shared resource $\boldsymbol{R}$  “Obligation” ($\varepsilon_{j}>0$) is a separate process from costs & benefits |
| Wu *et al.* 2019 |  | Analyzed conditions for system collapse in 81 models of mutualism representing different combinations of saturating benefits due to interspecific density-dependence; zero, linear, or saturating costs due to interspecific density-dependence; and effects of external stress. |  |
| Martignoni *et al.* 2020 | S37  S38 | $\left\{ \begin{aligned} \frac{d\boldsymbol{N}_{1}}{dt}=\boldsymbol{N}_{1}\left( r_{p}+\frac{q_{hp}\alpha\boldsymbol{N}_{2}}{d+\boldsymbol{N}_{1}}-q_{cp}\beta\boldsymbol{N}_{2}-\mu_{p}\boldsymbol{N}_{1} \right) \\ \frac{d\boldsymbol{N}_{2}}{dt}=\boldsymbol{N}_{2}\left( q_{cm}\beta\boldsymbol{N}_{1}-\frac{q_{hm}\alpha\boldsymbol{N}_{1}}{d+\boldsymbol{N}_{1}}-\mu_{m}\boldsymbol{N}_{2} \right) \end{aligned} \right.$ | Plant ($\boldsymbol{N}_{1}$)-Mycorrhizae ($\boldsymbol{N}_{2}$) |
| Hale *et al.* 2021 | S8 | $\frac{d\boldsymbol{N}_{2}}{dt}=\boldsymbol{N}_{2}\left( b_{2}+\varepsilon\frac{a\boldsymbol{N}_{1}}{1+ah\boldsymbol{N}_{1}}-{s_{2}\boldsymbol{N}_{2}-d}_{2} \right)$ | Set of models for Plant ($\boldsymbol{N}_{1}$, below)-Animal ($\boldsymbol{N}_{2}$) transport mutualisms  Obligate when $b_{2}-d_{2}\leq0$ |
|  | S39 | $\frac{d\boldsymbol{N}_{1}}{dt}=\boldsymbol{N}_{1}\left[ b_{P}\left( f+\varphi\frac{a\boldsymbol{N}_{2}\boldsymbol{N}_{1}}{1+ah\boldsymbol{N}_{1}+a\boldsymbol{N}_{2}\boldsymbol{N}_{1}} \right)g-s_{P}\boldsymbol{N}_{1}-d_{P} \right]$ | Pollinators increase seed set  Obligate when $b_{1}fg-d_{1}\leq0$ |
|  | S40 | $\frac{d\boldsymbol{N}_{1}}{dt}=\boldsymbol{N}_{1}\left[ b_{P}fg-\left( s_{P}-\sigma\frac{a\boldsymbol{N}_{2}}{1+ah\boldsymbol{N}_{1}+a\boldsymbol{N}_{2}} \right)\boldsymbol{N}_{1}-d_{P} \right]$ | Dispersers reduce negative density-dependence  Facultative only |
|  | S41 | $\frac{d\boldsymbol{N}_{1}}{dt}=\boldsymbol{N}_{1}\left[ b_{P}f\left( g+\gamma\frac{a\boldsymbol{N}_{2}}{1+ah\boldsymbol{N}_{1}+a\boldsymbol{N}_{2}} \right)-s_{P}\boldsymbol{N}_{1}-d_{P} \right]$ | Dispersers increase germination  Obligate when $b_{1}fg-d_{1}\leq0$ |
